# Exploring the Potential of Biomimetic Peptides in Targeting Fibrillar and Filamentous Alpha-Synuclein—An In Silico and Experimental Approach to Parkinson’s Disease

**DOI:** 10.3390/biomimetics9110705

**Published:** 2024-11-18

**Authors:** Sophia A. Frantzeskos, Mary A. Biggs, Ipsita A. Banerjee

**Affiliations:** Department of Chemistry and Biochemistry, Fordham University, 441 East Fordham Road, Bronx, NY 10458, USA; sfrantzeskos1@fordham.edu (S.A.F.); mbiggs2@fordham.edu (M.A.B.)

**Keywords:** alpha-synuclein, fibrillation, peptides, antioxidant, molecular dynamics

## Abstract

Alpha-synuclein (ASyn) is a protein that is known to play a critical role in Parkinson’s disease (PD) due to its propensity for misfolding and aggregation. Furthermore, this process leads to oxidative stress and the formation of free radicals that cause neuronal damage. In this study, we have utilized a biomimetic approach to design new peptides derived from marine natural resources. The peptides were designed using a peptide scrambling approach where antioxidant moieties were combined with fibrillary inhibition motifs in order to design peptides that would have a dual targeting effect on ASyn misfolding. Of the 20 designed peptides, 12 were selected for examining binding interactions through molecular docking and molecular dynamics approaches, which revealed that the peptides were binding to the pre-NAC and NAC (non-amyloid component) domain residues such as Tyr39, Asn65, Gly86, and Ala85, among others. Because ASyn filaments derived from Lewy body dementia (LBD) have a different secondary structure compared to pathogenic ASyn fibrils, both forms were tested computationally. Five of those peptides were utilized for laboratory validation based on those results. The binding interactions with fibrils were confirmed using surface plasmon resonance studies, where EQALMPWIWYWKDPNGS, PYYYWKDPNGS, and PYYYWKELAQM showed higher binding. Secondary structural analyses revealed their ability to induce conformational changes in ASyn fibrils. Additionally, PYYYWKDPNGS and PYYYWKELAQM also demonstrated antioxidant properties. This study provides insight into the binding interactions of varying forms of ASyn implicated in PD. The peptides may be further investigated for mitigating fibrillation at the cellular level and may have the potential to target ASyn.

## 1. Introduction

Parkinson’s disease (PD) is a neurodegenerative disease that is characterized by defects in motor activity, including resting tremor and muscular rigidity, as well as nonmotor symptoms, such as cognitive changes, dementia, and sleep disorders [1]. In particular, the degeneration and loss of dopaminergic neurons from the substantia nigra region of the brain has been implicated in the presentation of motor symptoms in PD [2,3]. While both genetic and environmental factors have been implicated, the loss of neurons is often accompanied by the formation of misfolded toxic proteinaceous inclusions called Lewy bodies [4]. The aggregation of these proteinaceous inclusions involves the interactions of several proteins, including synphilin-1, α-synuclein, parkin and ubiquitin C-terminal hydrolase L1 (UCH-L1) [5,6,7]. Of these, one of the major proteins implicated in the progression of PD is alpha-synuclein (ASyn) [8]. While not fully understood, the main function of ASyn has been linked to the control of neurotransmitter function and the neuromuscular system [9], and its misfolding and loss of function results in deleterious effects. In addition, PD progression has also been linked to oxidative stress due to free radical damage [10]. Approximately 5–10% of Parkinson’s cases are associated with mutations in PARK genes, which increase oxidative stress [11]. Mutations in SNCA, a PARK gene that encodes alpha-synuclein, have also been implicated in the early onset of PD [12].

Several reports have indicated the detection of copious amounts of ASyn oligomers and fibrils in Lewy bodies in the early stages of PD [13,14,15,16]. Neurons associated with oligomeric ASyn have been reported to have a higher level of oxidative stress. Furthermore, ASyn oligomers have been shown to induce ROS production and lower the levels of reduced glutathione [17]. Additionally, ASyn oligomers produce superoxide radicals by binding to transition metal ions such as copper and iron [18], and iron overload has also been implicated in oxidative stress, given its ability to modify ASyn upon binding. [19,20]. Furthermore, oxidative stress can also affect ASyn toxicity and mediate PD pathogenesis [21,22]. Thus, several studies are being conducted to elucidate the protein misfolding process of ASyn, and investigate ways to mitigate oxidative stress and misfolding. For example, to impede oxidative stress, curcumin, a natural antioxidant, has been recommended as a candidate drug for the potential treatment of PD, as it may replenish glutathione levels [23,24]. Several other natural antioxidants, such as lipoic acid, melatonin, carnitine, selenium, and natural polyphenols, as well as vitamins A, C, and E, have also been found to delay the progression of PD by reducing ROS levels [25]. In another study, researchers have shown that antioxidant nanoparticles prepared by the self-assembly of ferulic acid and tannic acid components with adipic acid as a linker reduced alpha-synuclein aggregation and lowered pro-inflammatory cytokines [26]. Although the administration of levodopa in conjunction with carbidopa or amantadine is one of the current mainstay treatments for PD [27,28,29], several other approaches are also being investigated. Furthermore, hydrogen-inhalation therapy has been shown to decrease activated microglia and pro-inflammatory cytokines, particularly in the case of levodopa-induced dyskinesia [30]. Monoamine oxidase type B inhibitors, such as rasagiline and selegiline, have been developed to enhance the use of dopamine by neurons [31]. However, the continued administration of dopamine-related drugs has been linked to a relative loss of efficacy over time [32].

More recently, peptide-based therapeutics have gained traction [33,34,35,36] due to their specificity and reduced toxicity. Current peptide-based approaches include utilizing brain-gut peptides that have been shown to have neuroprotective effects [37], including the glucagon-like peptide-1 receptor agonist GLP-1. It has been shown to protect against neurotoxin damage to the dopaminergic system in mice [38], restore levels of neurotransmitters depleted in PD, and alter cellular production and accumulation of amyloid beta (Aβ) deposits [39,40]. In another study, NPT-100-18a, a cyclic peptidomimetic compound derived from the peptide KKDQLGK, which interacts with membrane-bound Asyn, has been shown to disrupt and reduce the formation of oligomers in lipid membranes and reduce aggregation [41]. Small molecule disaggregators of ASyn and prion-like protein aggregates such as Anle138b [3-(1,3-benzodioxol-5-yl)-5-(3-bromophenyl)-1H-pyrazole] have also been developed [42,43,44].

Previous studies have also utilized computational methods such as molecular docking and molecular dynamics (MD) simulations to investigate the stability and binding interactions of various targets of PD. For example, the neuroprotective effects of the flavonoid karanjin were evaluated using molecular docking and molecular dynamics for five targets of Alzheimer’s disease (AD) and four targets of PD [45]. Docking scores showed comparatively higher potency against AD and PD than standard drugs. Another study examined the effect of flavonoids, including morin, quercetin, and myricetin, on Asyn fibrils using MD simulations [46]. They identified that the flavonoids destabilized the beta-sheet structure of Asyn fibrils and changed their morphology, with myricetin inducing the highest disruption, opening the potential for their use as a therapeutic to destabilize ASyn fibrils.

In this work, we have designed new peptides and utilized a biomimetic peptide-based computational approach to investigate their interactions with pathogenic fibrillar and filamentous segments of ASyn. To do so, several marine bioactive peptides were initially evaluated. The novel peptides were then designed using a peptide scrambling approach [47], where antioxidant peptide motifs were conjugated with short sequences of fibril inhibitory motifs (FIMs). The selected antioxidant peptide segments were derived from natural sources such as mussels, ark shell scapharca subcrenata, or the C-terminal superoxide dismutase domain of Arthrospira Platensis [48,49,50]. Each of the peptides has been shown to display antioxidant activity. To design the peptides, we created mutants of those peptide motifs to potentially further enhance their antioxidant activities. The FIMs were then connected at the N- or C-terminal or both ends of selected antioxidant motifs in order to develop peptides that may potentially mitigate ASyn fibrillation. In previous work, the FIMs (DPNGS and ELAQM) were shown to inhibit the fibrillation of insulin [51]; however, these have never been tested in conjunction with other peptides against ASyn. In total, 14 peptide sequences known for their antioxidant activity were screened using the AnOxPePred web server (https://services.healthtech.dtu.dk/services/AnOxPePred-1.0/ (accessed on 15 December 2023)) to predict their free radical scavenging activities. Those peptides were then mutated to design 40 new peptides to investigate if their antioxidant activity could be enhanced (Supplementary Information Table S1). Based on those results, we created 20 new peptide sequences by attaching selected antioxidant motifs to FIMs. Upon the attachment of the FIMs, the new peptide sequences were again screened through AnOxPePred to ensure that the peptides demonstrated antioxidant properties (Supplementary Information Table S2). Based on the most optimal results, 12 of those peptides were selected and investigated for their potential to bind to pathogenic fibrillar and the Lewy body-derived segment of filamentous ASyn and subjected to molecular docking studies and MD simulations to examine the binding interactions of those peptides with alpha-synuclein. We chose to examine both fibrillar and filamentous ASyn, as previous studies have demonstrated that different strains of ASyn possess different secondary structures, which lead to diverse levels of toxicity and propagation properties [52] that may result from the exposure of different regions in their fibril structures [53].

To validate the computational results, we examined five of the designed peptides and explored their binding interactions with ASyn fibrils through surface plasmon resonance (SPR) analysis. Furthermore, the impact on secondary structures of ASyn fibrils was examined through circular dichroism (CD) spectroscopy, where treatment with all peptides showed a reduction in the antiparallel beta-sheets over time, which was also corroborated through Thioflavin-T assay. Overall, this work establishes a basis for investigating the impact of short antioxidant peptides combined with FIMs and their influence on structurally different ASyn structures. It provides the groundwork for understanding the mechanism of binding interactions and the conformational changes involved in α-synucleinopathies for developing drug candidates for the potential treatment of PD.

## 2. Materials and Methods

### 2.1. Computational Methods

#### 2.1.1. Peptide Design

ChemDraw (22.2.0) was utilized to design each peptide sequence. The structures were then imported to Chem 3D 22.2.0 and energy-minimized using the MM2 energy minimization option from Chem 3D. The structures were then saved and exported as .pdb files and visualized on PyMOL.

#### 2.1.2. AnOxPePred

The AnOxPePred 1.0 web server [54] was used to determine if the peptides designed in the study displayed potential antioxidant activity. The web server uses convolutional neural networks to determine the antioxidant properties of peptides. The web server does so by predicting the free radical scavenging (FRS) and ion-chelating properties based on a dataset of experimentally tested antioxidant and non-antioxidant peptides. In general, the FASTA sequence of each peptide was input into the web server, which was utilized to determine chelation scores and FRS scores.

#### 2.1.3. Aggrescan

To ensure that the peptides themselves had low aggregation propensity, the web server Aggrescan [55] was utilized to detect potential “hot spots” of aggregation-prone areas in the designed peptides. The predictions are based on calculations on an aggregation propensity scale for each amino acid (a3V), which are derived from experimental in vivo data and on assumptions about sequences that modulate protein aggregation. The a3V average is calculated, which gives a4V. The number of hot spots, as well as the total area of hot spots and average a4v, were recorded for each peptide. The program obtains the information based on a database of 57 amyloidogenic proteins, in which the region of “hot spots” are experimentally known.

#### 2.1.4. C-I-TASSER

To investigate the secondary structures of the designed peptides, the web server C-I-Tasser (Contact-guided Iterative Threading Assembly Refinement) was used [56]. This web server works to generate inter-residue contact maps using multiple deep neural network predictors. C-I-TASSER uses a multi-threading approach (LOMETS) from structural templates obtained from PDB. The secondary structure, designated by coils (C), strands (S), and helices (H), as well as C-Score (confidence score) and TM-Score (template modeling score) values, were recorded for each designed peptide.

#### 2.1.5. Protein Processing

The .pdb files of the alpha-synuclein filament segments (derived from Lewy body dementia brains (PDB ID: 8a9l) [57]) and alpha-synuclein fibrils (PDB ID: 2n0a) [58] were downloaded from the RCSB database. Each file was opened on PyMOL (2.5.2) [59]. The files were cleaned by removing water molecules and any other bound ligands or molecules. The final structures were exported as .pdb files to be used in further analysis.

#### 2.1.6. SiteMap

In order to identify the binding regions for each form of alpha-synuclein, the Maestro Application SiteMap (from Schrodinger, New York, NY, USA) [60,61] was utilized. To identify the top-ranked binding sites, the number of site points was set to 15 per reported site, and the site maps were cropped at 4A° from the nearest site point.

#### 2.1.7. Molecular Docking Studies

Molecular docking studies were conducted for the 12 designed peptides with two forms of alpha-synuclein (PDB ID: 8a9l), which represents ASyn filaments derived from human brains with Lewy body formation and the pathological form of alpha-synuclein fibrils (PDB ID: 2n0a), using AutoDock Vina 1.2.0 [62], which uses gradient optimization and multi-threading [63]. The ligand-ASyn complexes were prepared using AutoDock Tools 1.5.6, where polar hydrogens were added along with Kollman charges. The files were then saved as .pdbqt files. In a different workspace window, the ligand previously prepared on ChemDraw 3D and PyMOL was saved as a .pdbqt file. Then, the specific ASyn and ligand files were opened in the same workspace where a grid box (40 × 40 × 40) Å with grid co-ordinates for each receptor was created. The grid co-ordinates for the PDB ID: 8a9l complexes were center x = 80.6; y = 95.4 and z = 98.0, and for PDB ID 2n0a, the grid co-ordinates were x = 116.1; y = 141.8 and z = −4.7. The binding affinities for each complex were recorded, and the results of the docking studies were visualized using PyMOL.

#### 2.1.8. Protein-Ligand Interactions Profiler (PLIP)

Each ASyn-ligand complex .pdb file obtained from AutoDock Vina was exported into Protein-Ligand Interaction Profiler (PLIP). This web server identifies the interactions and the residues involved [64,65]. The interaction results were visualized using PyMOL. The results obtained were tabulated to obtain the number and types of interactions involved, which included hydrogen bonds, hydrophobic interactions, salt bridges, π-π stacking interactions, or π-cation interactions.

#### 2.1.9. Molecular Dynamics (MDs) Simulations

MD simulations were carried out using DESMOND from Schrodinger [66,67]. This software is used to study biological systems with high performance and accuracy, using specific advanced algorithms and numerical techniques. The ASyn-ligand complexes with the highest binding affinity from the docking studies were prepared in PyMOL by adding hydrogens. The file was then exported as a .mae file and opened in the software Maestro 2023-2 to further prepare the peptide-ASyn complex. First, the ASyn proteins were prepared for each complex using the Protein Preparation Wizard. Hydrogens, missing side chains, and disulfide bonds were added as necessary. H-bonds were then optimized with a pH setting of 7.0. Each ligand (in this case, the designed peptide sequences) was then reincorporated with the prepared protein, and a solvated system was created using System Builder. We predefined the solvent model with SPC, used an orthorhombic box shape (10 × 10 × 10) Å, and minimized the volume of the box to best fit the complex. To best mimic physiological conditions, water was added to the box, and Na^+^ and Cl^−^ ions were added to neutralize the system. All systems were run locally on Maestro software 2023-2 from Schrodinger. Simulations were run using the OPLS4 force field [68] with a 100 ns trajectory, an NPT ensemble class, and conditions set at 310 K and 1.01325 bar. The resulting out.cms files were then opened in Maestro 2023-2 to be further analyzed using the Simulation Interaction Diagram tab. Trajectory images were gathered from each complex at different time points in order to visualize changes in binding interactions over time.

#### 2.1.10. MMGBSA Studies

Molecular Mechanics Generalized Born Surface Area (MMGBSA) energy calculations [69,70] were run for each complex in order to determine the theoretical free binding energies over the entire simulation. Trajectory files created from the molecular dynamics simulations were analyzed using the thermal_mmgbsa.py script, and the averages of free energy, electrostatic energy, H-bond energy, lipophilic energy, solvation, van der Waals, and pi-pi packing energy were recorded.

#### 2.1.11. Pharmacokinetics Prediction

The ADMETlab2.0 web server [71,72] was utilized in order to predict the absorption, distribution, metabolism, and excretion (ADME) study propensities of each of the designed peptides. The SMILES code of each peptide was uploaded for screening. Properties such as Pfizer rule acceptance, logP value, MDCK Cell Permeability, hERG Blocker, Pgp inhibitor/substrate, and blood-brain barrier permeability were recorded.

### 2.2. Laboratory Methods

#### 2.2.1. Surface Plasmon Resonance (SPR) Studies

In order to validate the results obtained from computational studies, five of the optimal peptides were utilized to confirm binding interactions with ASyn fibrillar aggregates (Abcam, Waltham, MA, USA). SPR provides information about the binding affinities of ligands with proteins in real time [73]. Gold-plated chips (Platypus Technologies, Madison, WI, USA) were soaked in ethanol in six-well plates and placed under UV light for 10 min. Ethanol was aspirated from the wells, followed by the addition of a layer of 11-mercaptoundecanoic acid (0.1 M) in ethanol to the gold side of the chips; this was incubated at 4 °C for 1 h to functionalize the chips [74]. After incubation, three drops of 0.1 M N-hydroxysiccinimide (NHS) and 1-Ethyl-3-(3-Dimethylaminopropyl) carbodiimide hydrochloride (EDAC), (0.1 M) were added to each chip and incubated for 2 h at 4 °C. Then, the protein solution of ASyn fibrillar aggregates (2 mg/mL) was added to each chip and incubated at 4 °C for 24 h. The coated chips were then spotted with one drop of Cargille’s 7.21 refractive index fluid and inserted into the SPR instrument (GWC Horizon SPR Imager II Instrument). Each run was calibrated with a 1X PBS solution until SPR intensity values were stable and less than or near equal to zero. Then, each of the peptide samples at varying concentrations (250 μM, 150 μM, 75 μM, 50 μM, and 25 μM) were injected into the SPR at a flow rate of 30 μL/min. Each run was repeated thrice, and the averages obtained were reported. The data obtained was input into GraphPad Prism 9 Software in order to calculate the KD values. Statistical analysis was carried out using student’s *t*-tests.

#### 2.2.2. Thioflavin-T (ThT) Assay

Thioflavin T assay was conducted to assess the effect of five peptides (PYYYWKELAQM; PYYYWKDPNGS; EQALMPEGMGLEDPNGS; ELAQMPYYYWKDPNGS; ELAQMPWIWYWKDPNGS) on ASyn preformed fibrils (StressMarq). Thioflavin T is a dye that, upon binding to *β* sheet conformation of peptides, exhibits a strong increase in fluorescence emission, making it a suitable sensor to study fibrillation kinetics in vitro. [75]. The samples were prepared by incubating 20 μM of ASyn diluted in PBS buffer with 20 μM of each peptide. To each sample, 70 μM ThT dye diluted in deionized water was added. The prepared solutions were then transferred into quartz cuvettes, and the emission spectra at different time intervals were recorded. Samples were excited at 440 nm, and emission spectra were recorded between 460 nm to 550 nm at a slit of 3 nm.

#### 2.2.3. Circular Dichroism (CD) Spectroscopy

CD measurements were carried out using a JASCO J-1500 spectrophotometer (Singapore) to examine the secondary structural changes of ASyn that occurred upon incubation with each of the peptides [76]. Each sample was run five times at the rate of 200 nm min^−1^ with a 0.5 nm step, 1 nm bandwidth, and then averaged. In general, 20 μM of each peptide was incubated with ASyn fibrils (20 μM) in PBS. A blank with PBS buffer was subtracted from the experimental spectra for correction. Further, the CD spectra of the neat peptides were measured, and the spectra of the peptides alone were subtracted from the samples containing ASyn incubated with peptides. Then, 190–250 nm range spectra were used for analysis. All the spectra were smoothed and converted to the mean residue ellipticity [*θ*] in deg ∗ cm^2^/dmol. The secondary structural analysis of each of the samples containing ASyn treated with peptides was examined by uploading the .txt files obtained from CD studies to the BeStSel (beta structure selection) web server. BeStSel uses reference CD spectra of known protein structures and estimates the different structural elements. [77,78].

#### 2.2.4. DPPH Antioxidant Assay

To investigate the antioxidant activity of the five peptides (PYYYWKELAQM; PYYYWKDPNGS; EQALMPEGMGLEDPNGS; ELAQMPYYYWKDPNGS; ELAQMPWIWYWKDPNGS), we conducted a colorimetric DPPH (2,2-diphenyl-1-picrylhydrazyl) radical scavenging assay using an antioxidant assay kit (AbCam, USA). In general, the protocol from the assay kit was followed to determine the antioxidant activity. Four different concentrations of each of the peptides were utilized (2 µM, 10 µM, 20 µM, and 40 µM). A standard curve was generated using Trolox, and then the activities of each of the peptides were compared by calculating the inhibition ratio.

## 3. Results and Discussion

### 3.1. Antioxidant Activity Prediction

Given the fact that oxidative stress has been known to impact ASyn misfolding and insinuate fibrillar structures and Lewy body inclusions, we designed peptides with potential antioxidant activities, as predicted by AnOxPePred studies. Furthermore, to examine if combining antioxidant peptide sequences with fibril-reducing motifs would impact binding with ASyn fibrils and ASyn filaments and affect conformations, we incorporated FIMs with the antioxidant peptides. As mentioned earlier, the FIMs in previous work have been shown to reduce insulin fibrillation. To determine the potential antioxidant properties of the 12 peptides, we utilized the AnOxPePred web server. The results are shown in Table 1. The antioxidant portions of the peptide segments are highlighted in blue, while the FIMs are highlighted in yellow. Overall, the FRS values ranged from 0.53 to 0.73. The sequence ELAQMPYYYWKDPNGS was predicted to show the highest FRS score. This peptide contains the FIM sequences ELAQM at the N-terminal and DPNGS at the C-terminal, while the PYYYWK was the antioxidant motif designed by mutating the known antioxidant sequence PIIVYWK, derived from the blue mussel *Mytilus edulis*. We replaced two of the isoleucine residues with tyrosine residues while also removing the valine residue, as tyrosine residues are known to demonstrate antioxidant activity [79]. In general, of the 12 sequences, 10 of the antioxidant motifs contained variations of the sequence PIIVYWK, where the isoleucine residue and/or the valine residue was replaced by tyrosine or tryptophan or both, with a goal of enhancing antioxidant activity [80]. Two of the sequences contained the antioxidant motifs GPEGPMGLE and GFYGPTE. The GPEGPMGLE sequence is derived from the collagen hydrolysates of red fish scales [81] and was shown to have strong radical scavenging activity, whereas the GFYGPTE sequence was obtained by mutating the GFIGPTE sequence, which is another antioxidant peptide derived from the collagen hydrolysate of red fish scales. As before, we replaced the isoleucine residue with tyrosine, which showed a higher FRS score, and we therefore utilized the mutated sequence.

### 3.2. C-ITASSER Studies

To determine the secondary structural elements of the 12 designed peptides, we conducted C-ITASSER studies. The results are shown in Table 2. In general, the predicted C-Score (confidence score) values are in the range of −5 to +2 [82]. This score is based on the significance of the LOMETS threading alignments, the satisfaction rate of the predicted contact maps, and the decoy convergence degree of the C-I-TASSER simulations. The highest C-Score among the peptides was found for the PIWWYWKDPNGS sequence at −0.56, and the lowest was ELAQMPYYYWKDPNGS at −1.89. The results were obtained from calculations based on the sequence profiles derived from the databases. The top 10 threading templates were selected to determine the secondary structure. The results predicted a variety of structures across the peptides, with some being composed of all coils (C), while others were a mix of either coils (C) and strands (S) or coils and helices (H). In addition, the template modeling (TM) scores, which lie in a range of 0 to 1, serve to provide a comparison of the similarities between predicted and template structures. The results obtained ranged from 0.49 to 0.64, with ELAQMGPEGPMGLEDPNGS and PIWWYWKDPNGS having the lowest and highest scores, respectively.

### 3.3. AGGRESCAN Studies

To ensure that the designed peptides show a low propensity of aggregation, we conducted studies using the web server AGGRESCAN. This web server is utilized to predict the aggregation potential in peptides based on the aggregation propensity scale for natural amino acids. It was used to determine “hot spots” of aggregation in each of the 12 peptides. The results are shown in Table 3, which indicates the number of hot spots in the sequence, total area, and total hot spot area for each amino acid in the sequence. The number of hot spots in the sequences ranged from 0 to 1, with peptides PYYYWKDPNGS, ELAQMPYYYWKDPNGS, and ELAQMGPEGPMGLEDPNGS showing zero hot spots. The total area and total hot spot area ranged from −7.373 to 3.522 and 0 to 4.075. The peptides that had the lowest and highest total areas were ELAQMGPEGPMGLEDPNGS and PIWWYWKELAQM, respectively, while peptides PYYYWKDPNGS, ELAQMPYYYWKDPNGS, and ELAQMGPEGPMGLEDPNGS had the lowest hot spot area while EQALMPWIWYWKDPNGS had the highest hot spot area. These results indicate that, overall, the propensity of the peptides to aggregate is predicted to be low.

### 3.4. SiteMap Analysis

**Figure 1:**
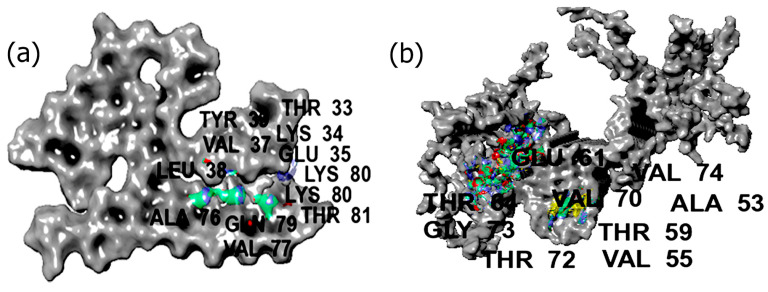
SiteMap analysis showing binding pocket regions in (**a**) Lewy body dementia-derived alpha-synuclein filament and (**b**) pathogenic alpha-synuclein fibrils. The explicit regions within the binding pocket are color-coded as follows: Hydrophilic regions—green; hydrophobic—yellow; hydrogen bond donor region—blue; H-bond acceptor region—red.

SiteMap analysis provides important insight into the binding regions in proteins [83]. As can be seen in Figure 1, the binding regions for filamentous ASyn from Lewy bodies were primarily observed across residues ranging from Thr33 to Leu38 and Ala76 to Ly80. In previous work, several regions of ASyn have been shown to have a higher propensity for aggregation, particularly the region containing residues of the non-Aβ component of amyloid plaques (NAC region), which includes amino acids 61–95, and tends to be more hydrophobic [84]. Additional regions susceptible to aggregation include residue regions 1–18 and 27–56 [85]. Thus, it appears that the regions displayed by the SiteMap analysis encompass the aggregation-prone regions in the case of filamentous ASyn. Interestingly, in the case of fibrillar ASyn, the binding pocket region was also found to be between VAL55 and GLY73, once again indicating aggregation-prone regions.

### 3.5. Molecular Docking Studies

To examine if the designed peptides were found to bind to ASyn, molecular docking studies were conducted with both forms of ASyn. The results are shown in Table 4.

As can be seen, the sequence PYYYWKELAWM showed similar binding affinities with both forms ASyn at −6.3 kcal/mol, while the highest binding affinity was seen for the EQALMGFYGPTEDPNGS sequence with fibrillar ASyn. The sequence PIWWYWKDPNGS showed the highest binding affinity with filamentous ASyn at −6.4 kcal/mol.

Interestingly, the sequences ELAQMPIWWYWKDPNGS and DPNGSPYYYWKELAQM both showed higher binding affinities with fibrillar ASyn at −5.7 kcal/mol and at −5.3 kcal/mol compared to that observed with ASyn filamentous from Lewy bodies at −4.8 kcal/mol and −4.4 kcal/mol, respectively.

#### 3.5.1. PLIP Analysis

To further delve into the binding interactions of the peptides with the two forms of ASyn, the web server PLIP was used to identify the specific interactions.

##### Interactions of Designed Peptides with Lewy Body Dementia (LBD) Filament

**Figure 2:**
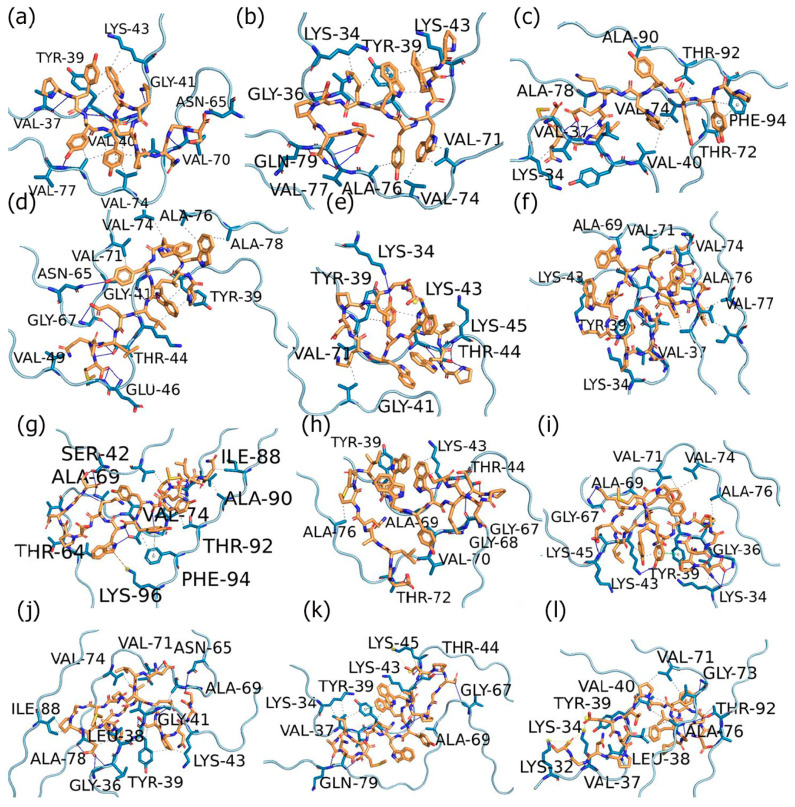
PLIP analysis for peptides with alpha-synuclein filaments derived from Lewy body dementia. (**a**) PYYYWKDPNGS; (**b**) PIWWYWKDPNGS; (**c**) PYYYWKELAQM; (**d**) PIWWYWKELAQM; (**e**) PWIWYWKDPNGS; (**f**) EQALMPWIWYWKDPNGS; (**g**) ELAQMPYYYWKDPNG; (**h**) ELAQMPIWWYWKDPNGS; (**i**) DPNGSPYYYWKELAQM; (**j**) DPNGSPIWWYWKELAQM; (**k**) ELAQMGPEGPMGLEDPNGS; (**l**) EQALMGFYGPTEDPNGS.

As seen in Figure 2 and Table S3 (Supplementary Information), the PYYYWKDPNGS and PIWWYWKDPNGS sequences interacted with residues in the regions between Lys34–Lys43 and Val71–Gln79. The PYYYWKDPNGS peptide also formed H-bonds with Asn65, while Tyr39 demonstrated stacking interactions with the PIWWYWKDPNGS sequence. Interestingly, the residues between Glu34 and Lys45 and Val63–Val95 of LBD fibrils are known to form a beta-sheet core structure [86,87]. The interactions of the peptides in this region are, thus, promising, as they may lead to a change in conformation in this region. Overall, a higher number of hydrophobic interactions compared to H-bonds were observed. In comparison, the PYYYWKELAQM sequence interacted with the Ala90–Phe94 region along with the Lys34–Val40 region and with residues Thr72, Val74, and Ala78. The hydrophobic interaction with Thr72 is particularly consequential, as upon mutation to Met72, Asyn aggregation increases [88]. Furthermore, Thr72 and Thr75 are known to play a crucial role in stabilizing ASyn fibrils and are, therefore, considered a therapeutic target. [89]. The PIWWYWKELAQM sequence showed a higher number of H-bond interactions (11) in a similar region, while hydrophobic interactions were seen with Tyr39, Val78, Ala76, Val71, Ala69, Lys43, and Val74. The PWIWYWKDPNGS sequence differs from the PIWWYWKDPNGS sequence in the position of the isoleucine residue; thus, many of the residues involved in binding were similar. However, for PWIWYWKDPNGS, a new salt bridge was seen with Lys43, while the stacking interaction with Tyr39 was not seen, though it was involved in three hydrophobic interactions. Another notable difference was that most of the interactions were seen in the Lys34 through Lys45 region, while only one interaction was seen between the NAC residue Val71.

The EQALMPWIWYWKDPNGS, ELAQMPYYYWKDPNGS, and ELAQMPIWWYWKDPNGS peptides also demonstrated similar interactions to those seen for the peptides described earlier. However, the ELAQMPYYYWKDPNGS (ELS) sequence showed unique H-bonds with Ser42, while Lys96 formed a pi-cation interaction. Additionally, stacking interactions with Phe94 and hydrophobic interactions with Ile88 and Ala90 were seen. Notably, ELAQMPIWWYWKDPNGS formed three H-bonds with Thr72, while ELAQMPYYYWKDPNGS formed two H-bonds, and EQALMPWIWYWKDPNGS interacted with Gly73 through a H-bond. Overall, a number of ASyn residues between residues 36–42 implicated in the aggregation of ASyn were involved in interacting with these peptides [90].

For the DPNGSPYYYWKELAQM and DPNGSPIWWYWKELAQM sequences, several common interactions were observed, such as those with residues Lys34, Tyr39, Lys43, Lys45, and the NAC region residue Ala69. Interestingly, DPNGSPIWWYWKELAQM formed a salt bridge with Lys45. Additionally, it showed H-bond interactions with NAC region residues Val66, Ala78, and Gln79 and hydrophobic interaction with Ala69, while DPNGSPYYYWKELAQM showed five hydrophobic interactions, including three with Val71, one each with Ala76 and Ala69, and one H-bond was formed with Gly67.

For the ELAQMGPEGPMGLEDPNGS and EQALMGFYGPTEDPNGS sequences, the position of the glutamine residue in the N-terminal varied along with the antioxidant motif region, while the C-terminal region (DPNGS) was maintained. While both peptides showed the same number of hydrogen bonds (11), EQALMGFYGPTEDPNGS showed 10 hydrophobic interactions compared to ELAQMGPEGPMGLEDPNGS, which showed nine. Interestingly, while EQALMGFYGPTEDPNGS formed salt bridge interactions with Lys34 and with Lys32, ELAQMGPEGPMGLEDPNGS did not show any interactions with those residues. In fact, ELAQMGPEGPMGLEDPNGS interacted with the residues in the region between Gly36–Gly41 and with Lys43, as well as with multiple residues from the NAC region. On the other hand, EQALMGFYGPTEDPNGS showed interactions with Ala91 and Thr92, along with Val74, Ala76, Gly73, and Val71, while no interactions were seen with Ile88. Overall, these results indicate that the peptides were binding to aggregation-prone regions near the N-terminal of ASyn as well as with the central NAC region residues. Though there were some common interactions, some of the peptides showed unique interactions that may be further utilized to investigate their impact on the structural aspects of ASyn filaments from Lewy body dementia (LBD).

#### 3.5.2. Interactions of the Designed Peptides with Pathogenic ASyn Fibrils

**Figure 3:**
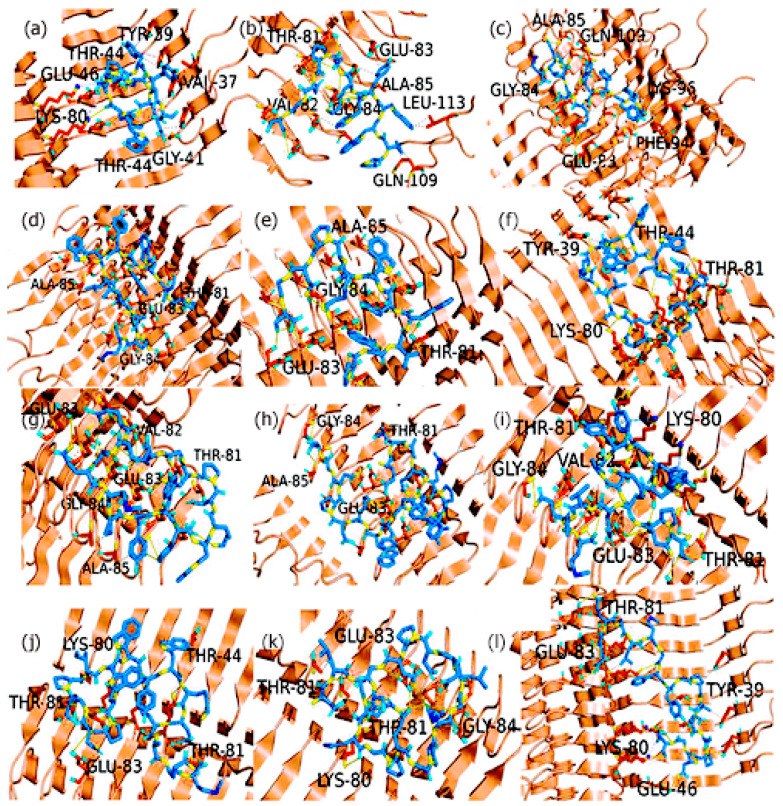
PLIP analysis for peptides with normal alpha-synuclein fibrils. (**a**) PYYYWKDPNGS; (**b**) PIWWYWKDPNGS; (**c**) PYYYWKELAQM; (**d**) PIWWYWKELAQM; (**e**) PWIWYWKDPNGS; (**f**) EQALMPWIWYWKDPNGS; (**g**) ELAQMPYYYWKDPNG; (**h**) ELAQMPIWWYWKDPNGS; (**i**) DPNGSPYYYWKELAQM; (**j**) DPNGSPIWWYWKELAQM; (**k**) ELAQMGPEGPMGLEDPNGS; (**l**) EQALMGFYGPTEDPNGS.

The ASyn fibril structure studied (PDB ID: 2n0a) constitutes a β-serpentine model with a Greek-key-β-sheet topology derived from a pathogenic form of ASyn fibrils. Given the many structural variations in different strains of ASyn fibrils, this model provides further insights into the binding interactions of the designed peptides with another form of ASyn. The PLIP analysis results (Figure 3) and Table S4 (Supplementary Information) show distinctly different residues involved in the interactions compared to those seen for the LBD ASyn filaments. Interestingly, most of the H-bond and hydrophobic interactions seen with the peptide sequences PIWWYWKDPNGS, PYYYWKELAQM, PIWWYWKELAQM, PWIWYWKDPNGS, ELAQMPYYYWKDPNG, ELAQMPIWWYWKDPNGS, DPNGSPYYYWKELAQM, DPNGSPIWWYWKELAQM, and ELAQMGPEGPMGLEDPNGS occurred with NAC region residues encompassing Lys80 through Lys86. This is likely because the ASyn structure contained exposed surfaces on the β-sheets around residues 82–86, while a majority of the NAC region was inaccessible. In addition, PIWWYWKDPNGS displayed hydrophobic interactions with the Leu113 and Gln109 of ASyn fibrils. Peptides PYYYWKDPNGS, EQALMPWIWYWKDPNGS, and EQALMGFYGPTEDPNGS showed interactions with both the NAC region residues and the pre-NAC region residues, though PYYYWKDPNGS predominantly interacted with residues Ser42, Glu46, Tyr39, Val37, and Thr44; the only interaction seen with the NAC region was seen with Lys80. The EQALMPWIWYWKDPNGS sequence formed four H-bonds with Lys80 and also showed multiple interactions with Thr81 and Glu83 residues. Similarly, EQALMGFYGPTEDPNGS demonstrated three H-bonds (one each) with Thr81, Glu83, and Lys80, along with two salt bridges with Lys80 and one hydrophobic interaction (each) with Thr81 and Lys80.

Thus, it appears that these three peptides bind differently compared to the peptides described earlier. This is particularly promising because the region in the β-sheet residues 68–82 has been implicated in fibril formation [91,92]. Other studies identified a similar hydrophobic 12-amino-acid region, residues 71 through 82, as essential for Asyn filament assembly and linked to neurotoxic properties associated with Asyn [93,94,95]; therefore, these are considered target regions for developing therapeutics. These results indicate that the designed peptides are binding to the identified toxic regions of Asyn. This is ideal for a potential therapeutic aimed at mitigating the misfolding of ASyn fibrils.

### 3.6. Molecular Dynamics Simulations

To further comprehend the binding interactions between the 12 peptides and the two forms of alpha-synuclein, we carried out MD simulations. The average root mean square deviation (RMSD) of three independent simulations for each complex over 100 ns can be seen in Table 5. In general, the values obtained for the filament-bound complexes were lower compared to those obtained for the fibrils. This is likely because of the single filament ASyn utilized, which has a smaller structure [96,97] compared to the ASyn fibrillar structure, which is more complex and comprised of multiple chains. The lowest RMSD values were seen upon complexation of the EQMPWIWYWKDPNGS and the ELAQMGPEGPMGLEDPNGS sequences with ASyn filament structure, while EQALMGFYGPTEDPNGS demonstrated the lowest average RMSD values with the fibrillar structure, followed by DPNGSPIWWYWKELAQM. Both PYYYWKDPNGS and PIWWYWKELAQM also showed the formation of relatively stable complexes with the ASyn filament structures. Based on the docking analysis, binding may have occurred with multiple residues from the pre-NAC and NAC regions, resulting in stable complexes in the case of the filament, while the fibrils, in most cases, were found to bind to the NAC region.

**Figure 4:**
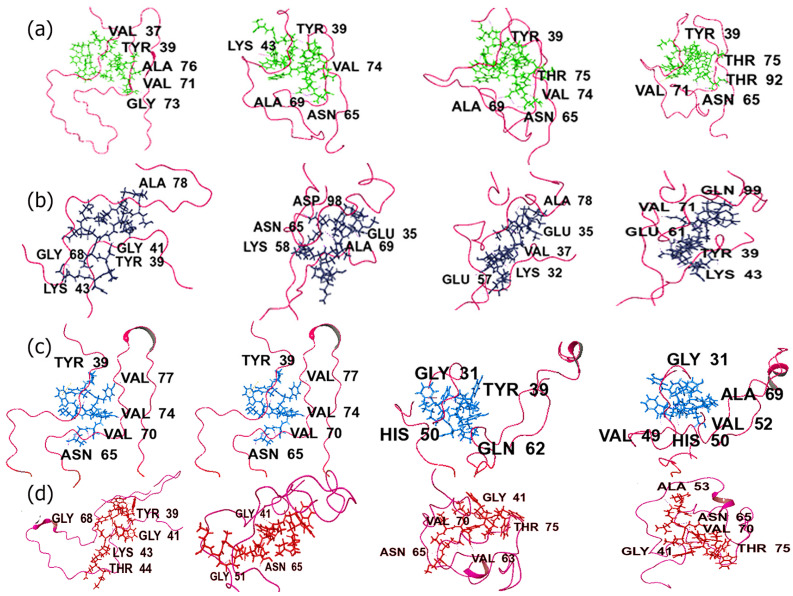
Trajectory images over 100 ns MD simulations with ASyn LBD-derived filament with (**a**) EQALMPWIWYWKDPNGS (green), (**b**) ELAQMGPEGPMGLEDPNGS (dark blue), (**c**) PYYYWKDPNGS (light blue), and (**d**) PIWWYWKELAQM (brown).

To further confirm the binding interactions of these complexes during the simulation, the trajectory snapshots were analyzed at various time points. Figure 4 shows the trajectory images with four of the peptides bound to the filament, including those that showed the lowest RMSD values. The trajectories for all other peptide-ASyn filament complexes are shown in Supplementary Information Figure S1. As can be seen in the case of the complexes formed with EQALMPWIWYWKDPNGS and the ELAQMGPEGPMGLEDPNGS, the peptides remained firmly attached to key residues within the aggregation-prone regions in the pre-NAC region, as described earlier (Tyr39, Val37), as well as NAC residues Thr75, Val84, Val73, and Thr92. Both peptides appeared to fold up and became more compact while maintaining binding interactions with the key residues. Interestingly, in the case of ELAQMGPEGPMGLEDPNGS, over the course of the simulation, interactions were also seen with the C-terminal domain residue Gln99. No major conformation change was observed within the structure over the simulation period, with ASyn largely demonstrating a coiled structure. For the PYYYWKDPNGS peptide complex, initially, while most interactions occurred with residues from the NAC region, with the exception of Tyr39, toward the end of the simulation, more interactions were seen with pre-NAC region residues, including Val49, His50, Gly31, and Val52. The only NAC residue interaction was with Ala69.Interestingly, the ASyn itself also appears to undergo a conformation change where in the appearance of alpha helical structure is seen toward the end. This is promising, indicating that this peptide not only binds to the peptide but also induces the formation of helical structures. The PIWWYWKELAQM peptide appears to remain firmly attached to the ASyn filament throughout the simulation. Key interactions were initially found in the region between Tyr39–Thr44 and with Gly68. However, after 75 ns, new interactions were seen with Val70, Asn65, and Thr75, which remained throughout the rest of the simulation, in addition to Gly41.

As shown in Supplementary Information Figure S1, the trajectory snapshots of the complex formed with PIWWYWKDPNGS display the appearance of beta-strand structures during the course of the simulation, accompanied by changes in the conformation of the peptide itself. While interactions with residues such as Tyr39 and Ser42 remained consistent throughout the simulation, new interactions were seen with Ser87 and Lys43 by the end of the simulation. The sequence PYYYWKELAQM, on the other hand, appears to induce the formation of helical turns by the end of the simulation when complexed with ASyn filament, showing interactions with Tyr30, Thr75, Lys43, and Gly41 throughout the simulation. Additionally, the peptide itself appears to undergo changes in conformation and moves away from the binding region. Interestingly, PWIWYWKDPNGS remains firmly attached to the pre-NAC region of the filament between residues Tyr39–Lys43 throughout the simulation. A new interaction is seen with Thr65 at the end of the simulation. Sequences ELAQMPYYYWKDPNGS and ELAQMPIWWYWKDPNGS appeared to bind more toward the NAC region residues as well as with pre-NAC residues throughout the simulation. Both of those peptides remained firmly attached to the ASyn filament, though ELAQMPIWWYWKDPNGS appeared to fold up more intricately within the ASyn filament. DPNGSPYYYWKELAQM, on the other hand, was found to show enhanced binding interactions over time, with residues such as Ser42, Glu57, Thr54, Thr44, and Glu46 at the end of the simulation. DPNGSPIWWYWKELAQM also displayed interactions within a similar region, in addition to a new interaction with the NAC region residue Thr72 by the end of the simulation. EQALMGFYGPTEDPNGS showed a wide range of interactions up to 50 ns into the simulation, including residues such as Thr92 and Gly93 along with Tyr39, Thr 72, and Ala76; however, after 75 ns, interactions were only seen with Ala76, Gly43, and Lys43. Additionally, the peptide remained firmly attached to the ASyn filament throughout the simulation.

**Figure 5:**
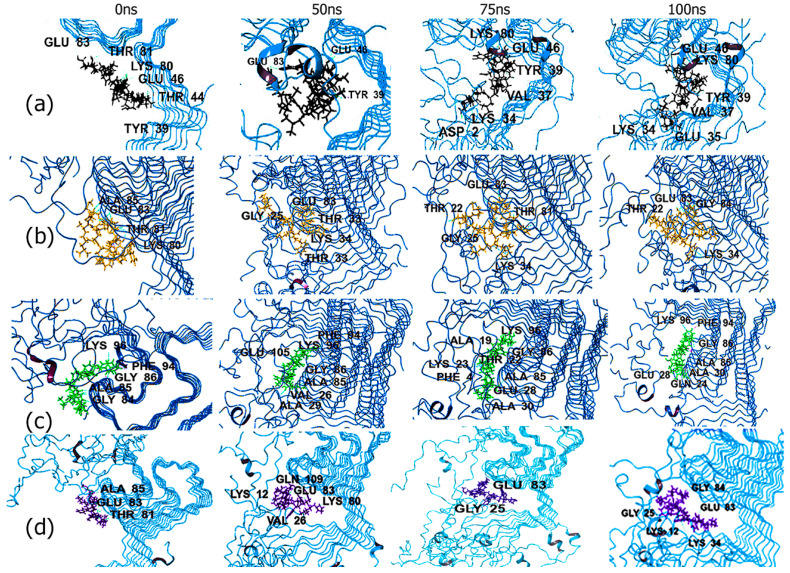
Trajectory images over 100 ns MD simulations with ASyn pathogenic fibrils. (**a**) EQALMGFYGPTEDPNGS (black); (**b**) DPNGSPYYYWKELAQM (yellow); (**c**) PYYYWKELAQM (green); (**d**) PIWWYWKELAQM (purple).

In the case of the trajectory images for fibrils, the four peptides that showed lower RMSD values are shown in Figure 5, and the remaining peptide complexes with ASyn fibrils are shown in Supplementary Information Figure S2. All four peptides (EQALMGFYGPTEDPNGS, (Figure 5a) DPNGSPYYYWKELAQM (Figure 5b), PYYYWKELAQM (Figure 5c), and PIWWYWKELAQM (Figure 5d) displayed interactions with residues within the central NAC domain, particularly in the range of residues Lys80–Gly86. Interestingly, in all cases, we observed that the peptides undergo conformation changes and, over the course of the simulation, interact with the N-terminal region residues ranging from Thr22–Ala30 in addition to residues from the NAC domain. This is particularly evident in the case of the PYYYWKELAQM sequence where, toward the end of the simulation, interactions are seen across the N-terminal domain residues Gln24, Glu28, and Ala30, as well as with the NAC domain residues Ala85, Gly86, and Phe94 and residue Lys96, which occurs at the beginning of the C-terminal domain of ASyn. These results indicate that the peptides are binding to multiple binding sites within ASyn and causing disruptions with the ASyn fibrils in some cases. Previous studies have shown that ASyn binds to membranes through the first 25 amino acid residues at higher concentrations of lipids [98], whereas at lower concentrations, the first 97 residues are said to be involved in binding to lipid membranes [99]. Furthermore, the appearance of helical structures can be seen (Figure 5b–d) during the course of the simulation. Previous work has reported that membrane binding induces ASyn to adopt helical structures locally [100]. Thus, it appears that these peptides may induce a similar conformation change within those binding regions of ASyn.

In the case of the complexes of PYYYWKDPNGS and PIWWYWKDPNGS (Figure S2a,b), conformation changes were observed in the ASyn fibrils, with the peptides moving deeper into the ASyn fibrillar chains over time. The PIWWYWKDPNGS peptide initially interacts with Thr81–Ala85; however, over the course of the simulation, interactions were seen with the N-terminal region residues such as Gly31 and Ala30. For the PYYYWKDPNGS complex, the peptide initially showed interactions with residues in the Tyr39–Glu46 region, along with a single interaction with Lys80; however, over time, the ligand was found to spread out within the ASyn fibrils, showing interactions with the N-terminal region residues such as Glu13, Glu20, and Ala 17 while still maintaining the interactions that were seen initially. Among the peptides PWIWYWKDPNGS, EQALMPWIWYWKDPNGS, and ELAQMPYYYWKDPNGS, over time, it appears that the sequence PWIWYWKDPNGS undergoes conformation changes, causing the fibrils to slightly detangle and form disordered structures; however, by the end of the simulation, the ligand appears to move outward while still maintaining interactions with residues such as Gly84, Glu83, and Lys34. EQALMPWIWYWKDPNGS initially showed changes in conformation within the first 50 ns, after which it appears to show very little changes, maintaining contact with residues in the NAC as well as pre-NAC regions by the end of the simulation. The sequence ELAQMPYYYWKDPNGS tends to fold up over the course of the simulation within the ASyn fibrils and remains firmly attached, maintaining key contacts with residues between Lys80–Ala85, as well as with N-terminal region residues such as Lys32, Thr33, Lys32, and Lys23, by the end of the simulation. The sequence ELAQMPIWWYWKDPNGS initially showed key interactions with NAC region residues; however, by the end of the simulation, it also interacted with the C-terminal region residues such as Asn103, Asp115, and Gln 109, in addition to the NAC region residues such as Glu83 and Ala85. The sequence ELAQMGPEGPMGLEDPNGS appears to demonstrate more stable binding with the ASyn fibrils when compared to DPNGSPIWWYWKELAQM. While ELAQMGPEGPMGLEDPNGS remains firmly attached throughout the simulation, this peptide also becomes more compact over time and interacts with residues of the NAC and C-terminal of ASyn fibrils, particularly between 50–100 ns of the simulation. DPNGSPIWWYWKELAQM, on the other hand, shows conformation changes over time and moves toward the far end of the N-terminal region, interacting with residues such as Gly7 and Ser9 while also interacting with Leu113 from the C-terminal of an adjacent ASyn chain. Additional interactions seen at the end of the simulation were those with Glu35 and Val37.

#### 3.6.1. Radius of Gyration (rGyr)Studies

The radius of gyration provides information about the compactness of the ligands upon binding to proteins [101]. As can be seen, for the filamentous ASyn (Supplementary Information Figure S3), the lowest rGyr is seen for the sequence PYYYWKDPNGS, followed by DPNGSPYYYWKELAQM, where the values remained relatively consistent (between 0.6 to 0.7 nm) throughout the simulation. While the rGyr value for PIWWYWKELQM was slightly higher (0.8 nm), the value remained consistent throughout the simulation. On the other hand, the peptide DPNGSPYYYWKELAQM showed a slight increase to 0.8 nm (from 0.69 nm) after 50 ns, which indicates that the peptide may spread out more after 50 ns but attains a stable conformation, as the value does not show significant changes after that. Similar behavior is observed for all peptides (values in the range of 0.8 nm to 0.9 nm), with the exception of EQALMGFYGPTEDPNGS and ELAQMPYYYWKDPNGS. In the case of EQALMGFYGPTEDPNGS, fluctuations in rGyr are seen over the first 50 ns; however, it appears to become more stable after 70 ns (at 0.9 nm to 0.95 nm). The least stable conformation was seen for ELAQMPYYYWKDPNGS, which showed continuous fluctuations over most of the simulation; however, at the end of the simulation, it appears to attain a stable conformation, as seen by a stable rGyr after 90 ns.

Interestingly, the PYYYWKDPNGS sequence also showed the most stable and lowest rGyr with the ASyn fibrillar structure, followed by PIWWYWKDPNGS and PIWWYWKELAQM. In general, the average rGyr values remained between 0.7 to 0.9 nm for all other peptides, indicating fairly stable conformation throughout the simulation. In the case of the ELAQPIWWYWKDPNGS sequence, the rGyr was found to attain lower values, particularly after 70 ns, which indicates that the peptide became more compact as the simulation progressed. The highest rGyr, with more fluctuations over the course of the simulation, was seen for the sequence EQALMGFYGPTDEDPNGS, which indicates that it formed the least compact complex with fibrillar ASyn. The DPNGSPIWWYWKELAQM sequence also showed fluctuations for the first 30 ns; however, the rGyr value was found to decrease and remained steady at 0.82 nm for the rest of the simulation. These results corroborate the RMSD behavior of the peptides discussed earlier.

#### 3.6.2. Root Mean Square Fluctuation

**Figure 6:**
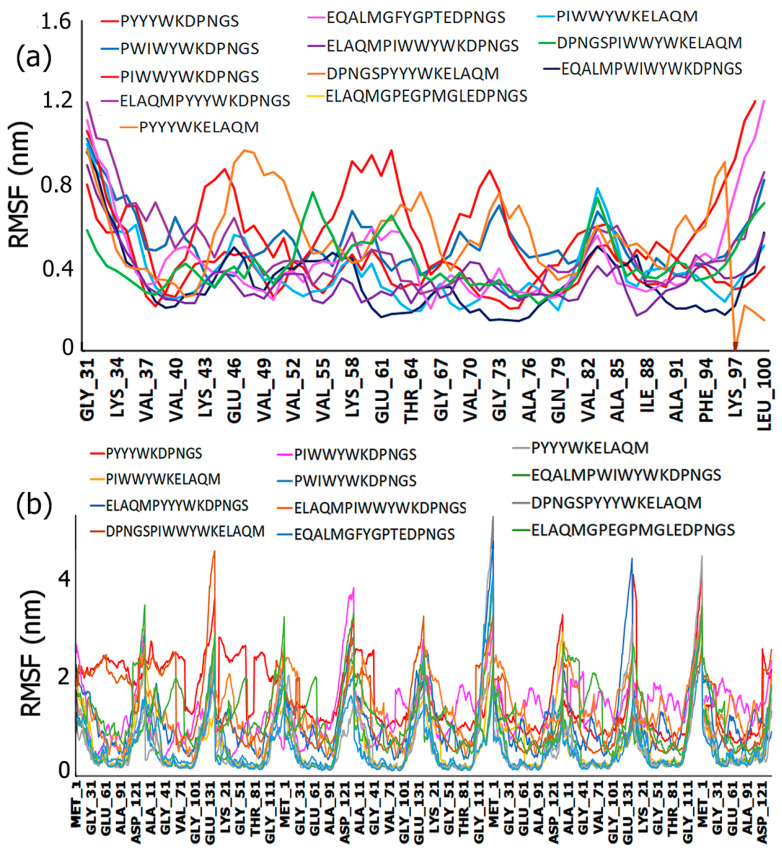
Comparison of root mean square fluctuation (RMSF) plots of the designed peptides with the (**a**) ASyn filament derived from Lewy body dementia brains and (**b**) pathogenic ASyn fibrils.

The RMSF values provide critical information about the changes in protein conformation and residues involved during the course of the simulation. The results of the RMSF values are shown in Figure 6. In general, in the case of ASyn filament (Figure 6a), fluctuations are observed across the entire protein during the simulation in all cases. Higher fluctuations are observed at the N- and C-terminal residue regions, as expected due to the higher flexibility of proteins in those regions [102]. Among the peptides, the sequence DPNGSPYYYWKELAQM demonstrated higher fluctuation compared to the other peptides, particularly in the region encompassing residues Lys45–Ala53, Thr63–Asn66, and Val71–Val77, implicating a higher probability of involvement of those residues in binding with the peptide. On the other hand, PIWWYWKDPNGS showed higher fluctuations in the region between residues Ala53–Val63 and Ala69–Gly73, which consist of the pre-NAC region and NAC region residues. These results are consistent with the docking studies, which revealed the involvement of those regions in binding with the peptides. Overall, the lowest RMSF values were seen for the ELAQMPIWWYWKDPNGS sequence upon binding, indicating that it is likely to have formed the most stable complex. All peptides also showed fluctuations in the region between Thr81 and Ser87, further implicating the role of the NAC region in binding with the peptides. In comparison, the neat (unbound) ASyn filament showed lower RMSF values in those regions.

The RMSF plot across multiple chains of ASyn fibrils is shown in Figure 6b. As shown, the higher RMSF peaks are seen across the regions encompassing the N- and C-terminal residues, particularly for peptides DPNGSPIWWYWKELAQM, PYYYWKELAQM, and ELAQMPYYYWKDPNGS. In addition, fluctuations were seen across the all ASyn protein segments, including the NAC and pre-NAC regions, indicating their involvement in binding with the peptides. Specifically, DPNGSPIWWYWKELAQM and PYYYWKELAQM exhibited higher interactions in those regions as well. The ELAQMGPEGPMGLEDPNGS sequence exhibited higher RMSF values between Glu46 and Val66 compared to other peptides. These results indicate that the peptides appear to interact with the fibrils in similar regions, albeit causing variable changes in flexibility. This is particularly important for targeting specific regions of ASyn to develop therapeutics.

#### 3.6.3. MMGBSA Analysis

The binding free energies (ΔGbind) for each of the peptides with both forms of ASyn were then calculated using MM-GBSA (molecular mechanics generalized born surface area continuum solvation) calculations. This methodology provides an estimation of the free energies of the binding of ligands with proteins over the entire simulation process [103]. The results are shown in Table 6. For both forms of ASyn, van der Waals interactions were the major contributor to binding, followed by coulomb forces. In the case of the ASyn filament, EQALMPWIWYWKDPNGS at −118.37 kcal/mol demonstrated the highest binding energy, followed by PIWWYWKELAQM at −115.66 kcal/mol and DPNGSPYYYWKELAQM at −109.28 kcal/mol. On the other hand, the Asyn fibrils demonstrated the highest binding energy with PYYYWKDPNGS at −109.79 kcal/mol, followed by ELAQMGPEGPMGLEDPNGS at −93.88 kcal/mol and PYYYWKELAQM at −77.28 kcal/mol. The weakest binding was seen for PIWWYWKELAQM at −57.85 kcal/mol, which is interesting, given that it displayed a significantly higher binding interaction with the monomeric filament. These results further confirm that the ligands involved in binding to the two forms of ASyn vary. This was expected, given the differences in the structures of ASyn studied, and indicates that the interactions with monomeric LBD-derived filament have a higher binding overall compared to the ASyn fibrillar aggregates.

### 3.7. Laboratory Validation Studies

#### 3.7.1. Surface Plasmon Resonance Studies

**Figure 7:**
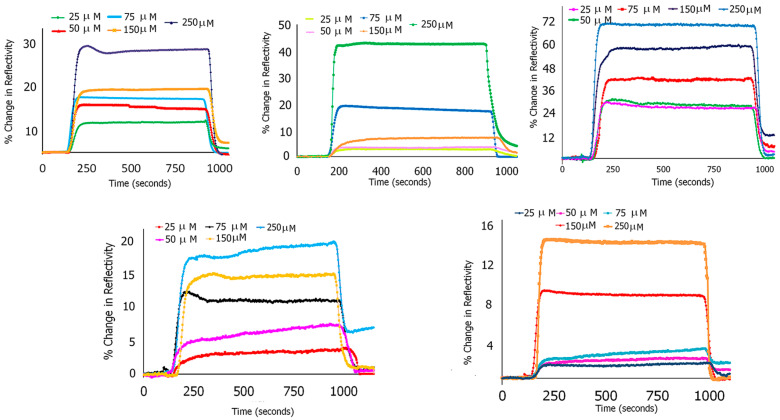
SPR sensograms showing binding with ASyn aggregates. Top row, from left to right: PYYYWKDPNGS; PYYYWKELAQM and EQALMPWIWYWKDPNGS. Bottom row, from left to right: ELAQPYYYWKDPNGS and ELAQPEGPMGLEDPNGS.

Based on computational studies, we selected five peptides that demonstrated efficient binding with ASyn fibrils or filaments. To validate the binding interactions, we conducted SPR analysis with fibrillar aggregates of ASyn as a proof of concept. In previous work, it has been shown that SPR analysis was utilized to examine the binding interactions of herbal extracts, such as Salix aegyptiaca, containing flavonoid moieties with ASyn [104] and efficiently demonstrated differences in binding interactions. The results obtained for the five peptides selected for lab validation studies are shown in Figure 7. In general, each of the peptides was found to bind to ASyn in a concentration-dependent manner. The peptide EQALMPWIWYWKDPNGS showed the lowest KD value (28.4 μM), followed by PYYYWKDPNGS at a KD value of 39.2 μM, while the KD value obtained for PYYYWKELAQM was found to be 55.2 μM. In comparison, ELAQMPYYYWKDPNGS and ELAQMGPEGMGLEDPNGS showed weaker binding, and the KD values were found to be 133.2 µM and 194.2 µM, respectively. Thus, both computational and SPR analysis confirm the high binding of EQALMPWIWYWKDPNGS and PYYYWKDPNGS with ASyn fibrils. Overall, these results confirm the binding of the peptides with ASyn fibrils.

#### 3.7.2. Thioflavin-T Assay

**Figure 8:**
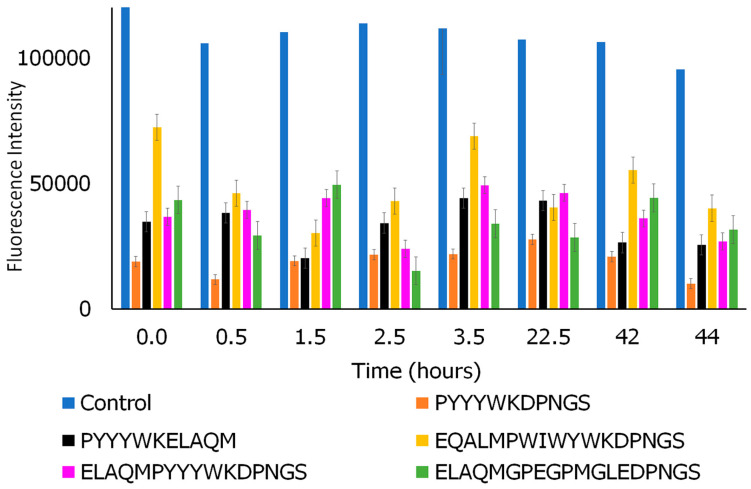
Comparison of fluorescence of thioflavin T over time for ASyn fibrils before (control) and after incubation with peptides.

A Thioflavin-T assay provides information about the changes in beta-sheet conformation, which are known to play a role in fibril formation [105]. To examine the impact of the peptides on ASyn fibrils, we conducted thioflavin-T assays. The results are shown in Figure 8. As can be seen, over the course of 46 h, changes in the fluorescence intensity of ASyn fibrils in the presence of each peptide can be observed. Overall, in the presence of each of the peptides, a reduction in fluorescence intensity was observed compared to neat untreated ASyn. This indicates that the peptides are not only binding to ASyn but also impacting the fibrillation process. Of the peptides, specifically, the PYYYWKDPNGS sequence demonstrated the highest change in fluorescence intensity, with a 46.2% reduction, while the ELAQMPYYYWKDPNS sequence showed a 27% reduction. Interestingly, EQALPWIWYWKDPNGS was found to show relatively higher fluorescence compared to those seen with other peptides; however, a gradual reduction in fluorescence intensity was observed (42%) at 48 h. On the other hand, ELAQMGPEGPMGLEDPNGS showed a 32% reduction over time. Thus, overall, it appears that the peptides can alter the conformation of the fibrils.

#### 3.7.3. CD Spectroscopy

**Figure 9:**
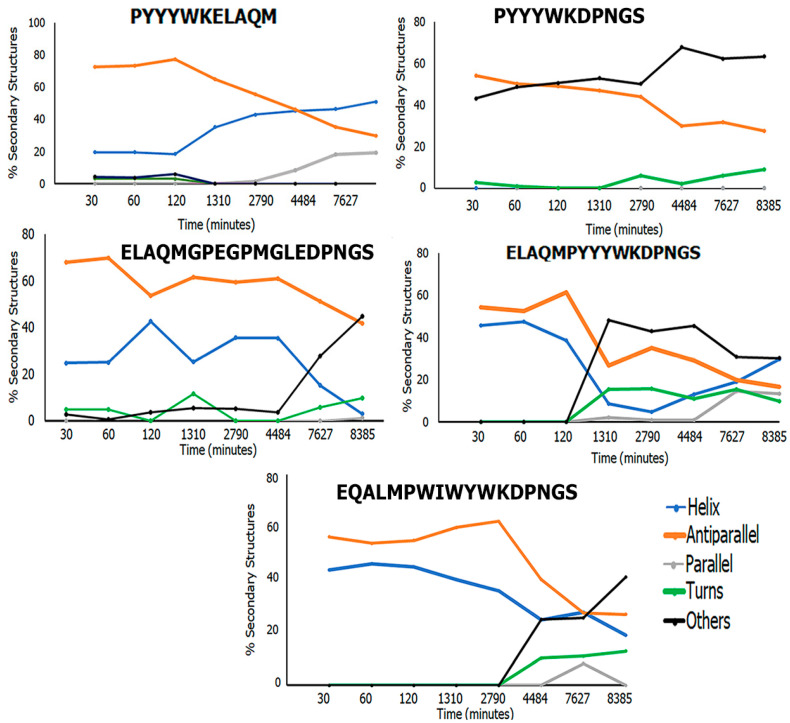
Comparison of the secondary structures of ASyn upon incubation with peptides.

To further elucidate the structural changes that occurred upon binding to the peptides, we conducted CD spectroscopic analyses. CD spectra were obtained at various times after incubating the peptides with ASyn fibrils for a period of 139 h (8385 min). The spectra were subtracted from those of the neat peptides. The average spectra obtained were then analyzed using the BeStSel web server, which provided information on the secondary structural changes that occurred over time [106]. The results obtained are shown in Figure 9. As can be seen, the results show that there is a decrease in antiparallel beta-sheet structures in all cases. Interestingly, in the presence of PYYYWKELAQM, conformational changes in ASyn resulted in the formation of alpha-helices and a gradual increase in parallel beta-sheets (20%). However, in the case of PYYYWKDPNGS, no alpha helices were seen; instead, the number of disordered structures increased over time. This suggests that the beta-sheet structures may be partially disrupted over time. In general, it is known that for beta-amyloids, antiparallel β-sheet oligomers rapidly form fibrils and are associated with mid-to-late-stage protein aggregation, while parallel β-sheets are slower in fibrillation [107]. Thus, it appears that PYYYWKELAQM may slow down the formation of fibrils, though it does not completely disrupt it compared to PYYYWKDPNGS, where antiparallel beta-sheets were reduced, and no parallel beta-sheets were seen. Among the ELAQMPYYYWKDPNGS, ELAQMPEGMGLEDPNGS, and EQALMPWIWYWKDPNGS treated samples, the largest decrease in the antiparallel beta-sheet structures was seen upon treatment with ELAQMPYYYWKDPNGS; this also showed a concomitant increase in turns and disordered structures and the appearance of parallel beta-sheet structures (20%) after 74 h, which subsequently diminished. Interestingly, all three of the treated samples also showed changes in the alpha-helical structures, with both ELAQMPEGMGLEDPNGS and EQALMPWIWYWKDPNGS showing an initial increase followed by a decrease in alpha-helical structures. ELAQMPYYYWKDPNGS showed an initial decrease followed by a gradual increase in alpha-helical structures. These results once again confirm that the peptides not only bind with ASyn but also induce conformational changes. Furthermore, the results corroborate those obtained from the thioflavin-T assay, which showed a decrease in beta-sheet structures with time, implying that the peptides may play a role in potentially reducing or arresting fibril formation, particularly for PYYYWKDNGS and ELAQMPEGMGLEDPNGS.

#### 3.7.4. Assessment of Antioxidant Activity

**Figure 10:**
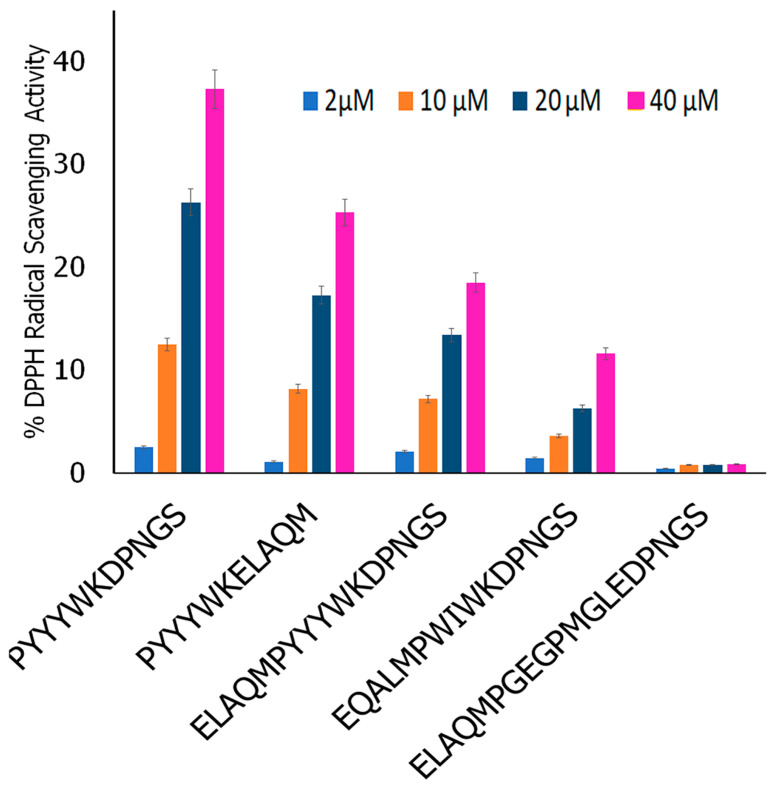
Comparison of DPPH radical scavenging activity of the peptides at varying concentrations.

To examine the antioxidant activity of the five peptides, we carried out DPPH assays. Previous studies have shown that DPPH assays are valuable for examining the antioxidant activity of a multitude of antioxidant compounds, including natural products [108]. The results obtained are shown in Figure 10. In comparison to Trolox (standard), the PYYYWKDNGS and PYYYWKELAQM sequences demonstrated 37% and 26% activity at the highest concentrations, respectively. Comparatively, EQALMPWIWYWKDPNGS showed 19% activity, while ELAQMPYYYWKDPNGS showed 12% activity. However, ELAQMGEPEGMGLEDPNGS showed negligible antioxidant activity. As can be seen, the first six amino acids of the PYYYWKDPNGS and PYYYWKELAQM sequences contain the antioxidant moieties, and the second half of the sequences contain the FIM moieties. EQALMPWIWYWKDPNGS, ELAQMPYYYWKDPNGS, and ELAQMGEPEGMGLEDPNGS, on the other hand, contain FIM moieties at both ends, with the antioxidant moiety sandwiched in between. Thus, =the antioxidant moiety may be less exposed compared to when it is at the N- or C-terminal. Interestingly, ELAQMGPEGPMGLEDPNGS, which was predicted to demonstrate a higher FRS score (0.59), showed the lowest DPPH radical scavenging activity, while the PYYYWKDPNGS and PYYYWKELAQM sequences showed higher activity. This could be attributed to the fact that the in vitro studies herein involved examining DPPH radical scavengers, whereas the FRS scores are calculated using models that are trained on a dataset comprising experimentally tested antioxidant and non-antioxidant peptides. The types of free radicals tested may vary.

## 4. Prediction of Pharmacokinetic Properties

The web server ADMETlab2.0 was used to predict the pharmacokinetic properties of all 12 of the designed peptides. The results obtained are shown in Table 7. All peptides were accepted by the Pfizer rule (Lipinski’s rule of five). This rule aims to assess druglike behavior, including physiochemical properties, aqueous solubility, permeability, and oral bioavailability [109,110]. The predicted logP values represent the predicted partition coefficient for each of the peptides between the aqueous and lipophilic phases. In general, the logP values varied from −5.150 to 0.579, with ELAQMGPEGPMGLEDPNGS having the lowest score and PYYYWKELAQM having the highest score. This is likely due to the presence of several negatively charged residues in the case of ELAQMGPEGPMGLEDPNGS, with a pI of 2.87, while PYYYWKELAQM demonstrated a pI value of 6.85 (near neutral). The MDCK cell permeability values indicate that the peptides are generally expected to permeate the cellular membrane. The highest permeability was found for EQALMGFYGPTEDPNGS at 4.2 × 10^−6^, and the lowest was found for ELAQMPYYYWKDPNGS at 9 × 10^−7^. Furthermore, the peptides were not found to be hERG blockers, indicating that these will not cause cardiotoxicity related to hERG channel inhibition [111]. The likelihood of the peptides acting as P-glycoprotein (Pgp) or multidrug-resistant protein 1 inhibitors or substrates was also predicted. As can be seen, none of the peptides were predicted to be Pgp inhibitors. In addition, several of the peptides (with the exception of EQALMGFYGPTEDPNGS, ELAQMGPEGPMGLEDPNGS, DPNGSPYYWKELAQM, EQALMPWIWYWKDPNGS, and PIWWYWKDPNGS) were also predicted to demonstrate very little activity as Pgp substrates, which indicates that the likelihood of these peptides activating PgP and driving efflux is low [112]. The web server was also used to predict blood-brain barrier (BBB) permeability. All peptides, with the exception of DPNGSPYYYWKELAQM and EQALMGFYGPTEDPNGS, were found to be BBB permeable, which is promising.

## 5. Conclusions and Future Work

In this study, we utilized a peptide scrambling approach to design 20 biomimetic peptides that comprised both antioxidant and fibrillary inhibition moieties. Of those, 12 peptides were then selected for investigating their antioxidant activity, secondary structures, and binding abilities with two forms of ASyn (pathogenic fibrillar and Lewy body dementia-derived filaments) using computation methods. Our results indicate that the peptides showed free radical scavenging scores between 0.53 and 0.73, with higher values predicted to show higher antioxidant activity. The molecular docking and MD simulations revealed key interactions with the NAC domain as well as with the pre-NAC domain and the appearance of alpha-helical structures over time, particularly with PYYYWKELAQM and ELAQMPYYYWKDPNGS. The docking studies revealed that, in general, the peptides bound to pivotal residues in the NAC domain, including residues such as Ala78, Val77, Ile88, and Gly73, among others. The molecular dynamics simulations further showed stable binding with most peptides, which was further confirmed by MMGBSA analysis, showing a ΔGbind energy between −80 kcal/mol and −118 kcal/mol for the peptides upon binding with the filamentous Lewy body-derived ASyn. Two peptides (PIWWWYWKDPNGS and PYYYYWKELAQM), however, showed a relatively lower ΔGbind (between −50 and −58.7 kcal/mol). On the other hand, PYYYWDKPNGS showed the highest ΔGbind with multichain fibrillar ASyn at −101.8 kcal/mol, while DPNGSPIWWYWKELAQM showed the lowest binding energy at −57.6 kcal/mol. Thus, the binding interactions varied between the filamentous and fibrillar ASyn. Based on the computational results, five peptides were selected for laboratory validation studies. In general, we observed that the peptides induced conformation changes in the ASyn fibrils. The results corroborate the MD simulations. Furthermore, antiparallel beta-sheets were found to reduce over time, with the concurrent appearance of disordered structures and turns, while some of the peptides also showed changes in alpha-helical content. In some cases, the appearance of parallel beta-sheet structures was observed, though to a lesser extent. The results were also confirmed by using thioflavin-T assays, which showed a reduction in fluorescence due to fibrillar ASyn over time. Overall, it appears that PYYYWKDPNGS and ELAQMPYYYWKDPNGS demonstrated both antioxidant activity and a reduction in beta-sheets over time and may be potentially utilized for further investigation into laboratory studies or conjugated with drugs that may mitigate the fibrillation of alpha-synuclein as a potential therapeutic. This work proposes a number of peptides that can be investigated to examine their impact on alpha-synuclein misfolding. Further studies will involve investigating the impact of the peptides in a cellular environment where the internalization of these peptides will be studied, as well as the impact on intra and extracellular ASyn fibrils.

## Figures and Tables

**Table 1:** Free Radical Scavenger Scores and Chelation Scores for newly designed peptides containing antioxidant motifs fused with FIMs, obtained using AnOXPePred.

Peptide Sequence	FRS Score	Chelation Score
PYYYWKDPNGS	0.53	0.14
PYYYWKELAQM	0.53	0.16
PWIWYWKDPNGS	0.53	0.15
PIWWYWKDPNGS	0.55	0.16
PIWWYWKELAQM	0.56	0.18
DPNGSPIWWYWKELAQM	0.58	0.19
ELAQMGPEGPMGLEDPNGS	0.59	0.23
EQALMGFYGPTEDPNGS	0.61	0.18
EQALMPWIWYWKDPNGS	0.68	0.15
DPNGSPYYYWKELAQM	0.68	0.18
ELAQMPIWWYWKDPNGS	0.69	0.15
ELAQMPYYYWKDPNGS	0.73	0.13

**Table 2:** Results of C-ITASSER web server predictions for each of the designed peptides.

Peptide	Secondary Structure	C-Score	TM-Score
PIWWYWKDPNGS	CCCSSSCCCCCC	−0.56	0.64 ± 0.13
PYYYWKDPNGS	CCCCCCCCCCC	−0.68	0.63 ± 0.14
DPNGSPIWWYWKELAQM	CCCCCCHHHHHHHHHHC	−1.14	0.57 ± 0.15
PWIWYWKDPNGS	CCSSSSCCCCCC	−1.25	0.56 ± 0.15
PIWWYWKELAQM	CHHHCCCCCCCCCCCCCCC	−1.34	0.55 ± −0.15
PYYYWKELAQM	CCHHHHHHHHC	−1.37	0.55 ± 0.15
EQALMPWIWYWKDPNGS	CCCCCCSSSSSSCCCCC	−1.47	0.53 ± 0.15
ELAQMPIWWYWKDPNGS	CCCCCCSSSSSSCCCCC	−1.52	0.53 ± 0.15
DPNGSPYYYWKELAQM	CCCCCCCHHHHHHHHC	−1.68	0.51 ± 0.15
EQALMGFYGPTEDPNGS	CCCCCCCCCCCCCCCCC	−1.71	0.51 ± 0.15
ELAQMGPEGPMGLEDPNGS	CHHHCCCCCCCCCCCCCCC	−1.84	0.49 ± 0.15
ELAQMPYYYWKDPNGS	CCCCCCCSSSSCCCCC	−1.89	0.49 ± 0.15

**Table 3:** AGGRESCAN web server predictions of the aggregation potential of designed peptides via the analysis of the number of hot spots, total area, and total hot spot area.

Peptide	Number of Hot Spots	Total Area	Total Hot Spot Area
PYYYWKDPNGS	0	−1.441	0
ELAQMPYYYWKDPNGS	0	−1.377	0
ELAQMGPEGPMGLEDPNGS	0	−7.373	0
PIWWYWKELAQM	1	3.522	3.79
PWIWYWKDPNGS	1	0.115	3.568
EQALMPWIWYWKDPNGS	1	1.266	4.075
PIWWYWKDPNGS	1	−0.042	3.411
ELAQMPIWWYWKDPNGS	1	0.221	3.918
DPNGSPYYYWKELAQM	1	−0.803	2.699
DPNGSPIWWYWKELAQM	1	0.795	4.056
PYYYWKELAQM	1	2.123	2.433
EQALMGFYGPTEDPNGS	1	−3.623	3.075

**Table 4:** Binding Affinity results obtained from molecular docking studies.

Peptide Sequence	ASyn Filament from Lewy Body Dementia (kcal/mol)	Pathogenic Fibrils of ASyn (kcal/mol)
PYYYWKDPNGS	−5.9	−5.2
PIWWYWKDPNGS	−6.4	−6.5
PYYYWKELAQM	−6.3	−6.3
PIWWYWKELAQM	−6.2	−5.7
PWIWYWKDPNGS	−5.0	−6.4
EQALMPWIWYWKDPNGS	−5.7	−5.8
ELAQMPYYYWKDPNGS	−5.2	−6.2
ELAQMPIWWYWKDPNGS	−4.8	−5.7
DPNGSPYYYWKELAQM	−4.4	−5.3
DPNGSPIWWYWKELAQM	−4.9	−4.4
ELAQMGPEGPMGLEDPNGS	−5.6	−5.2
EQALMGFYGPTEDPNGS	−5.1	−6.6

**Table 5:** Average RMSD values (nm) of the peptide complexes formed with ASyn filaments and fibrils.

Peptide	Average RMSD (nm) of Complex with ASyn Filament from LBD	Average RMSD (nm) of Complex with Pathogenic ASyn Fibrils
PYYYWKDPNGS	0.82	5.5
PIWWYWKDPNGS	1.6	1.7
PYYYWKELAQM	1.3	1.5
PIWWYWKELAQM	0.88	1.6
PWIWYWKDPNGS	1.3	1.9
EQALMPWIWYWKDPNGS	0.6	1.7
ELAQMPYYYWKDPNGS	1.0	10.3
ELAQMPIWWYWKDPNGS	0.74	5.3
DPNGSPYYYWKELAQM	1.6	1.1
DPNGSPIWWYWKELAQM	0.64	1.6
EQALMGFYGPTEDPNGS	1.0	0.9
ELAQMGPEGPMGLEDPNGS	0.6	3.7

**Table 6:** (**a**) MMBGBA analysis of designed peptides with ASyn filaments from LBD. (**b**) MMBGBA analysis of designed peptides with pathogenic ASyn fibrils.

Peptide	ΔG Bind kcalc/mol	Coulomb kcal/mol	H-Bond kcal/mol	Lipophilic kcal/mol	Solvent GB kcal/mol	Van der Waals kcal/mol
(**a**)						
EQALMPWIWYWKDPNGS	−118.37	−49.90	−5.94	−21.40	48.11	−87.29
PIWWYWKELAQM	−115.66	−53.13	−4.52	−22.59	42.89	−79.79
DPNGSPYYYWKELAQM	−109.28	−58.25	−4.88	−22.19	52.96	−79.94
ELAQMGPEGPMGLEDEPNGS	−104.86	−62.71	−7.97	−16.69	71.10	−88.72
ELAQMPYYYWKDPNGS	−98.66	−39.26	−4.64	−22.02	46.25	−80.24
DPNGSPIWWYWKELAQM	−93.40	−46.45	−5.64	−14.10	48.08	−72.38
EQALMGFYGPTEDPNGS	−91.48	−49.02	−4.35	−17.76	47.62	−72.52
ELAQMPIWWYWKDPNGS	−80.66	−32.79	−3.38	−15.17	32.18	−61.69
PIWWYWKDPNGS	−58.73	−27.65	−2.27	−12.62	25.21	−43.61
PYYYWKELAQM	−50.37	−32.35	−2.35	−8.82	31.23	−39.54
PWIWYWKDPNGS	−50.37	−32.35	−2.35	−8.82	31.23	−39.54
(**b**)						
**Peptide**	**ΔG Bind kcalc/mol**	**Coulomb kcal/mol**	**H-Bond kcal/mol**	**Lipophilic** **kcal/mol**	**Solvent GB** **kcal/mol**	**Van der** **Waals kcal/mol**
PYYYWKDPNGS	−101.79	−52.87	−9.27	−31.20	77.61	−89.06
ELAQMGPEGPMGLEDEPNGS	−93.88	−89.66	−9.76	−14.72	122.34	−105.08
DPNGSPYYYWKELAQM	−87.31	−63.77	−7.02	−13.83	95.05	−98.67
PYYYWKELAQM	−77.28	−35.73	−3.56	−22.24	59.92	−79.09
PIWWYWKDPNGS	−69.94	−39.80	−6.07	−11.35	67.89	−82.20
EQALMGFYGPTEDPNGS	−63.98	−41.39	−6.77	−16.72	73.31	−72.44
ELAQMPIWWYWKDPNGS	−63.75	−47.91	−3.90	−12.94	71.09	−73.97
ELAQMPYYYWKDPNGS	−63.33	−66.84	−8.46	−10.35	96.86	−77.19
PWIWYWKDPNGS	−62.07	−59.36	−6.38	−7.70	86.57	−76.25
EQALMPWIWYWKDPNGS	−60.76	−57.88	−5.32	−14.87	84.50	−66.36
PIWWYWKELAQM	−57.85	−60.02	−5.85	−12.61	96.43	−76.89
DPNGSPIWWYWKELAQM	−57.67	−40.97	−5.25	−15.19	81.25	−80.71

**Table 7:** Prediction of pharmacological properties (ADMET2.0lab).

Peptide Sequence	Pfizer Rule	LogP	MDCK Cell Permeability	hERG Blocker	PgP Inhibitor/ Substrate	Blood Brain Barrier Permeability
PYYYWKDPNGS	Accepted	−1.986	1.4 × 10^−6^	-	0/0.005	Yes
PIWWYWKDPNGS	Accepted	0.457	1.3 × 10^−6^	-	0.001/0.28	Yes
PYYYWKELAQM	Accepted	0.579	1 × 10^−6^	-	0/0.26	Yes
PIWWYWKELAQM	Accepted	3.195	2.3 × 10^−6^	-	0.02/0.95	Yes
PWIWYWKDPNGS	Accepted	0.457	1.3 × 10^−6^	-	0.001/0.28	Yes
ELAQMPWIWYWKDPNGS	Accepted	0.299	7.4 × 10^−7^	-	0/0.93	Yes
ELAQMPYYYWKDPNGS	Accepted	−2.146	9 × 10^−7^	-	0/0.26	Yes
ELAQMPIWWYWKDPNGS	Accepted	0.372	6.8 × 10^−7^	-	0/0.26	Yes
DPNGSPIWWYWKELAQM	Accepted	0.314	7.7 × 10^−7^	-	0/0.96	Yes
DPNGSPYYYWKELAQM	Accepted	−2.119	8.6 × 10^−7^	-	0/0.26	No
ELAQMGPEGPMGLEDPNGS	Accepted	−5.150	1.2 × 10^−6^	-	0/0.81	Yes
EQALMGFYGPTEDPNGS	Accepted	−4.049	4.2 × 10^−6^	-	0/0.81	No

## Data Availability

The original contributions presented in the study are included in the article and in Supplementary Materials.

## Supplementary Materials

The following supporting information can be downloaded at https://www.mdpi.com/article/10.3390/biomimetics9110705/s1, Table S1: Design of Antioxidant Peptides and their Free Radical Scavenging (FRS) scores. Table S2: Prediction of Free Radical Scavenger Scores of Designed peptides containing Antioxidant peptide motifs fused with fibrillary Inhibitory Motifs. Table S3: PLIP Analysis—Interactions of Designed Peptides with Filamentous ASyn derived from Lewy Bodies. Table S4: PLIP Analysis—Interactions of Designed Peptides with Pathogenic ASyn fibrils. Figure S1: Trajectory snapshots of peptide complexes with ASyn derived from LBD filaments over 100 ns simulations (a) PIWWYWKDPNGS; (b) PYYYWKELAQM (c) PWIWYWKDPNGS; (d) ELAQMPYYYWKDPNGS; (e) ELA QMPIWWYWKDPNGS; (f) DPNGSPIWWWYWKELAQM; (g) DPNGSPIWWYWKELAQM; (h) EQALMGFYGPTEDPNGS. Figure S2: Trajectory snapshots of peptide complexes with ASyn fibrils over 100 ns simulations (a) PYYYWKDPNGS; (b) PIWWYWKDPNGS; (c) PWIWYWKDPNGS; (d) EQALMPWIWYWKDPNG S; (e) ELAQMPYYYWKDPNGS; (f) ELAQMPIWWYWKDPNGS; (g) DPNGSPIWWWYWKELAQM; (h) ELAQMGPEGPMGLEDPNGS. Figure S3: Radius of gyration studies of designed peptides with (a) ASyn filament derived from LBD; (b) Pathogenic ASyn fibrils.
