# Supplementary material for: Exploring the Potential of Biomimetic Peptides in Targeting Fibrillar and Filamentous Alpha-Synuclein—An In Silico and Experimental Approach to Parkinson’s Disease
Source: Biomimetics (Basel). 2024 Nov 18;9(11):705. doi: 10.3390/biomimetics9110705 (PMC11591946; doi:10.3390/biomimetics9110705)
Supplement: Supplementary file 1 [file biomimetics-09-00705-s001.zip › biomimetics-3275219-supplementary.pdf]

Supplementary Information

Exploring the Potential of Biomimetic Peptides in Targeting Fibrillar and Filamentous Alpha-Synuclein – An In Silico and Experimental Approach for Parkinson's Disease

Sophia A. Frantzeskos[1], Mary A. Biggs[1] and Ipsita A Banerjee[1,*]
[1]*Department of Chemistry & Biochemistry, Fordham University, 441 E. Fordham Road, Bronx, New York 10458, USA*

Table S1. Design of Antioxidant Peptides and their Free Radical Scavenging (FRS) scores

Known Peptide Segment Derived from Natural Sources	Source	FRS Score	Mutant Sequences	FRS Score
HPLDSLCL	Ark Shell (*Scapharca Subcrenata*) [a]	0.3458	HPYDSYCY	0.5271
EPLSD	Marine bivalve mollusk (*tergillarca granosa)* [b]	0.3518	EPYSD	0.4586
			EPWSD	0.4464
WLDPDG	Marine bivalve mollusk (*tergillarca granosa)* [b]	0.4434	WYDPDG	0.5089
MDLFTE	Marine bivalve mollusk (*tergillarca granosa)* [b]	0.3668	MDYFTE	0.4682
WPPD	Marine bivalve mollusk (*tergillarca granosa)* [b]	0.5171	WYPD	0.5769
CYIE	Marine bivalve mollusk (*tergillarca granosa)* [b]	0.4324	CYYE	0.5691
LGLDVWEHAYYL	C-terminal SOD domain of *Arthrospira platens*is [c]	0.5743	LGWDVWEHAYYL	0.6055
			WGLDVWEHAYYL	0.5739
			LGLDVWEHAYYW	0.5735
			WGWDWWEHAYYW	0.6634
			WGWDVWEHAYYL	0.6045
			WGLDWWEHAYYL	0.6358
			WGLDVWEHAYYW	0.5732
PIIVYWK	blue mussel protein hydrolysate [f]	0.4370	PYYYWK	0.7131
			PIYVYWK	0.552
			PYIVWK	0.4824
			PWWVYWK	0.6125
			PWIWYWK	0.6319
			PWWWYWK	0.7155
			PIWWYWK	0.6461
FSVVPSPK	blue mussel protein hydrolysate [f]	0.3998	FSYYPSPK	0.5880
			FSYVPSPK	0.4727
			FSVYPSPK	0.5145
			FSWWPSPK	0.5606
MCLDSCLL	Ark Shell (*Scarpharca subcrenata*) [a]	0.3344	MCYDSCYY	0.4848
			MCWDSCLL	0.3385
			MCLDSCWL	0.3695
			MCLDSCLW	0.3336
			MCWDSCWL	0.4256
			MCWDSCLW	0.3897
			MCWDSCWW	0.4266
			MCLDSCWW	0.3705
EPVV	Marine bivalve mollusk (*tergillarca granosa)* [b]	0.3761	EPYY	0.6176
			EPVW	0.4741
			EPWV	0.4929
			EPWW	0.5934
VECYGPNRPQF	Algae protein waste hydrolysate [d]	0.5597	WECYGPNRPQF	0.5605
GFIGPTE	Collagen hydrosylate of Redlip Croaker scales [e]	0.4425	GFWGPTE	0.5379
GPEGPMGLE	Collagen hydrosylate of Redlip Croaker scales [e]	0.5264	GPEGPMGWE	0.5278

[a] Jin J.E., Ahb C.B., Je J.Y. Purification and characterization of antioxidant peptides from enzymatically hydrolyzed ark shell (*Scapharca subcrenata*) *Process Biochem.* **2018**, *72*, 170–176;
[b] Purification and characterization of antioxidant peptides derived from protein hydrosylate of the marine bivalve mollusk Tergillarca granosa *Marine Drugs*, **2019**, *17*, 251.

[c] Sannasimuthu A.; Kumaresan V.; Pasupuleti M.; Paray B. A.; Al-Sadoon M. K.; Arockiaraj J. Radical scavenging property of a novel peptide derived from C-terminal SOD domain of superoxide dismutase enzyme in *Arthrospira platensis*. *Algal. Res.* **2018**, *35*, 519–529;
[d] Sheih I. C.; Wu T. K.; Fang T. J. Antioxidant properties of a new antioxidative peptide from algae protein waste hydrolysate in different oxidation systems. *Bioresour. Technol.* **2009**, *100*, 3419–3425;
[e] Wang, W-Y.; Zhao, Y-Q.; Zhao, G-X.; Chi, C-F.; Wang, B. Antioxidant peptides from collagen hydrosylate of redlip croaker (Pseudosciena Polyactis) Scales: Preparation, characterization, and cytoprotective effects on H2O2-Damaged HepG2 cells. *Mar. Drugs*. **2020**, *18*, 156;
[f] Park, S. Y.; Kim, Y-S.; Ahn, C-B.; Je, J-Y. Partial purification and identification of three antioxidant peptides with hepatoprotective effects from blue mussel (Mytilus edulis) hydrosylate by peptic hydrolysis. *J. Funct. Foods* **2016**, *20*, 88-95.

Table S2. Prediction of Free Radical Scavenger Scores of Designed peptides containing Antioxidant peptide motifs fused with fibrillary Inhibitory Motifs.

Designed Sequence Antioxidant motif + FIM motif	FRS Score
PYYYWKDPNGS	0.527
PIWWYWKDPNGS	0.553
PYYYWKELAQM	0.534
PIWWYWKELAQM	0.561
PIIVYWKDPNGS	0.458
PIIVYWKELAQM	0.466
PWIWYWKDPNGS	0.532
EQALMPWIWYWKDPNGS	0.679
PWIWYWKEQALM	0.529
ELAQMPYYYWKDPNGS	0.729
ELAQMPIWWYWKDPNGS	0.691
DPNGSPYYYWKELAQM	0.684
DPNGSPIWWYWKELAQM	0.585
ELAQMPIIVYWKDPNGS	0.489
DPNGSPIIVYWKELAQM	0.374
GPEGPMGLEDPNGS	0.411
GPEGPMGLEELAQM	0.435
ELAQMGPEGPMGLEDPNGS	0.588
GFYGPTEDPNGS	0.427
GFYGPTEELAQM	0.425
EQALMGFYGPTEDPNGS	0.606
DPNGS (FIM MOTIF ALONE)	0.392
ELAQM (FIM MOTIF ALONE)	0.375

Table S3. PLIP Analysis – Interactions of Designed Peptides with Filamentous ASyn derived from Lewy Bodies.

With: PYYYWKDPNGS

Hydrogen Bonds	Distance H-A (Å)	Hydrophobic Interactions	Distance (Å)	Salt Bridge Residues	Distance (Å)	Pi-stacking Residues	Distance (Å)
GLY 36	2.03	LYS 34	3.5	N/A	N/A	PHE 94	4.86
VAL 37	2.98	LEU 38	3.44				
VAL 37	2.03	VAL 40	3.51				
VAL 37	3.22	THR 72	3.89				
TYR 39	2.63	VAL 74	3.39				
ALA 78	2.32	VAL 74	3.88				
		ALA 90	3.96				
		THR 92	3.65				
		PHE 94	3.67				

With PIWWYWKDPNGS:

Hydrogen Bonds	Distance H-A (Å)	Hydrophobic Interactions	Distance (Å)	Salt Bridges	Distance (Å)	Pi-stacking	Distance (Å)
VAL 37	3.56	TYR 39	3.65	N/A	N/A	N/A	N/A
TYR 39	3.19	TYR 39	3.37				
GLY 41	2.45	VAL 40	3.76				
GLY 41	1.95	VAL 40	3.32				
ASN 65	1.99	LYS 43	3.42				
VAL 70	2.49	LYS 43	3.66				
VAL 70	1.82	VAL 71	3.14				
VAL 77	2.41	VAL 74	3.68				
		VAL 74	3.55				
		VAL 74	3.69				
		ALA 76	3.68				
		ALA 76	3.44				

With PYYYWKELAQM

Hydrogen Bonds	Distance H-A (Å)	Hydrophobic Interactions	Distance (Å)	Salt Bridges	Distance (Å)	Pi-stacking	Distance (Å)
GLY 36	2.57	LYS 34	3.44	N/A	N/A	TYR 39	4.08
VAL 37	2.05	TYR 39	3.64				
LYS 43	2.49	TYR 39	3.53				
VAL 77	2.48	TYR 39	3.8				
VAL 77	2.71	LYS 43	3.28				
ALA 78	3.5	LYS 43	3.91				
GLN 79	1.97	VAL 71	3.65				
		VAL 71	3.9				
		VAL 74	3.52				
		ALA 76	3.47				

With PIWWYWKELAQM

Hydrogen Bonds	Distance H-A (Å)	Hydrophobic Interactions	Distance (Å)	Salt Bridges	Distance (Å)	Pi-stacking	Distance (Å)
GLY 41	2.01	TYR 39	3.87	N/A	N/A	N/A	N/A

GLY 41	2.69	TYR 39	3.37
LYS 43	2.29	TYR 39	3.68
THR 44	2.79	LYS 43	3.58
THR 44	2.74	VAL 49	3.72
THR 44	3.20	ALA 69	3.82
GLU 46	2.36	VAL 71	3.89
GLY 47	2.14	VAL 74	3.95
ASN 65	3.02	ALA 76	3.71
GLY 67	2.25	ALA 78	3.69
GLY 67	3.02		

With PWIWYWKDPNGS

Hydrogen Bonds	Distance H-A (Å)	Hydrophobic Interactions	Distance (Å)	Salt Bridge Residues	Distance (Å)	Pi-stacking Residues	Distance (Å)
LYS 34	3.5	TYR 39	3.66	LYS 43	3.44	N/A	N/A
TYR 39	2.69	TYR 39	3.53				
GLY 41	3.12	TYR 39	3.62				
GLY 41	1.86	VAL 40	3.64				
LYS 43	2.49	LYS 43	3.75				
LYS 43	2.4	LYS 45	3.65				
LYS 43	3.2	VAL 71	3.65				
THR 44	2.71						
THR 44	3.16						
LYS 45	2.13						

With EQALMPWIWYWKDPNGS

Hydrogen Bonds	Distance H-A (Å)	Hydrophobic Interactions	Distance (Å)	Salt Bridge Residues	Distance (Å)	Pi-stacking	Distance (Å)
GLY 36	2.18	LYS 34	3.49	N/A	N/A	N/A	N/A
VAL 37	2.77	LEU 38	3.55				
VAL 37	3.41	TYR 39	3.49				
TYR 39	2.4	VAL 40	3.81				
TYR 39	2.28	LYS 43	3.67				
GLY 41	2.28	ALA 69	3.69				
GLY 73	2.57	VAL 71	3.6				
VAL 74	2.91	VAL 74	3.6				
ALA 76	3.06	ALA 76	3.51				
ALA 76	2.83	VAL 77	3.33				
		ALA 78	3.8				
		ILE 88	3.95				
		ALA 90	3.73				

With ELAQMPYYYWKDPNGS

Hydrogen Bonds	Distance H-A (Å)	Hydrophobic Interactions	Distance (Å)	Pi-Cation Interactions	Distance (Å)	Pi-stacking	Distance (Å)
SER 42	2.67	VAL 40	3.51	LYS 96	5.83	PHE 94	4.89
SER 42	1.84	VAL 63	3.49				
THR 64	2.58	VAL 71	3.45				
VAL 66	2.64	THR 72	3.8				

ALA 69	2.55	VAL 74	3.62
ALA 69	3.39	ALA 76	3.34
VAL 71	2.12	VAL 77	3.55
VAL 71	2.38	ILE 88	3.57
THR 72	2.44	ALA 90	3.74
THR 72	2.29	THR 92	3.45
GLY 73	2.97	PHE 94	3.81
VAL 74	2		
ALA 76	2.88		

With ELAQMPIWWYWKDPNGS

Hydrogen Bonds	Distance H-A (Å)	Hydrophobic Interactions	Distance (Å)	Salt Bridges	Distance (Å)	Pi-stacking	Distance (Å)
TYR 39	3.24	TYR 39	3.9	N/A	N/A	N/A	N/A
TYR 39	2.15	LYS 43	3.38				
GLY 41	2.46	LYS 43	3.65				
LYS 43	3.07	ALA 69	3.87				
THR 44	2.74	ALA 76	3.52				
GLY 67	2.01						
GLY 67	1.99						
GLY 68	3.23						
GLY 68	2.48						
VAL 70	1.95						
THR 72	2.29						
THR 72	2.26						
THR 72	2.76						

With DPNGSPYYYWKELAQM

Hydrogen Bonds	Distance H-A (Å)	Hydrophobic Interactions	Distance (Å)	Salt Bridges	Distance (Å)	Pi-stacking	Distance (Å)
LYS 34	2.07	LYS 34	3.88	N/A	N/A	TYR 39	4.16
LYS 34	2.23	VAL 37	3.57				
GLY 36	2.38	LEU 38	3.67				
VAL 37	2.56	TYR 39	3.42				
LYS 43	2.4	LYS 43	3.57				
THR 44	2.11	LYS 43	2.67				
LYS 45	3	ALA 69	2.93				
GLY 67	2.7	VAL 71	3.63				
		VAL 71	3.6				
		VAL 74	3.73				
		ALA 76	4.0				

With DPNGSPIWWYWKELAQM

Hydrogen Bonds	Distance H-A (Å)	Hydrophobic Interactions	Distance (Å)	Salt Bridges	Distance (Å)	Pi-stacking	Distance (Å)
VAL 37	2.18	LYS 34	3.76	LYS 45	5.46	TYR 39	3.96
TYR 39	3.07	LYS 34	3.9				
GLY 41	1.95	VAL 37	3.71				
GLY 41	3.5	LEU 38	3.79				
LYS 43	2.01	TYR 39	3.97				

THR 44	3.16	TYR 39	3.49
THR 44	3.27	LYS 43	3.91
LYS 45	2.58	LYS 43	3.45
VAL 66	3.13	LYS 45	3.97
ALA 78	2.58	ALA 69	3.63
GLN 79	2.53		

With ELAQMGPEGPMGLEDPNGS

Hydrogen Bonds	Distance H-A (Å)	Hydrophobic Interactions	Distance (Å)	Salt Bridges	Distance (Å)	Pi-stacking	Distance (Å)
GLY 36	3.58	LEU 38	3.98	N/A	N/A	N/A	N/A
VAL 37	2.56	TYR 39	4				
TYR 39	1.82	TYR 39	3.5				
GLY 41	2.91	VAL 40	3.68				
GLY 41	2.62	LYS 43	3.71				
LYS 43	2.38	ALA 69	3.44				
ASN 65	3.39	VAL 74	3.76				
GLY 68	2.1	ILE 88	3.74				
VAL 71	2.3	ILE 88	3.75				
ALA 78	2.56						
ALA 78	3.02						

With EQALMGFYGPTEDPNGS

Hydrogen Bond Residues	Distance H-A (Å)	Hydrophobic Interactions	Distance (Å)	Salt Bridges	Distance (Å)	Pi-stacking	Distance (Å)
LYS 32	2.87	LEU 38	3.56	LYS 32	4.54	N/A	N/A
LYS 34	2.7	LEU 38	3.59	LYS 34	4.07		
LYS 34	1.96	LEU 38	3.37	LYS 34	3.99		
VAL 37	3.32	TYR 39	3.76				
TYR 39	2.45	VAL 40	3.96				
TYR 39	2.21	VAL 40	3.78				
GLY 73	2.33	VAL 71	3.73				
VAL 74	3.16	VAL 71	3.91				
ALA 76	2.99	ALA 76	3.82				
ALA 91	2.03	THR 92	3.68				
THR 92	2.03						

Table S4. PLIP Analysis – Interactions of Designed Peptides with Pathogenic ASyn fibrils.

With PYYYWKDPNGS

Hydrogen Bonds	Distance H-A (Å)	Hydrophobic Interactions	Distance (Å)	Salt Bridges	Distance (Å)	Pi-stacking	Distance (Å)
SER 42	2.94	VAL 37	3.67	LYS 80	3.47	TYR 39	4.75
GLU 46	3.34	TYR 39	3.73	LYS 80	5.49	TYR 39	5.13
GLU 46	2.22	TYR 39	3.66				
GLU 46	2.39	TYR 39	3.24				
LYS 80	2.76	TYR 39	3.55				
		THR 44	3.91				
		THR 44	3.96				

With PIWWYWKDPNGS

Hydrogen Bonds	Distance H-A (Å)	Hydrophobic Interactions	Distance (Å)	Salt Bridges	Distance (Å)	Pi-stacking	Distance (Å)
THR 81	2.47	GLU 83	3.56	N/A	N/A	N/A	N/A
THR 81	3.15	GLU 83	3.74				
THR 81	2.91	GLU 83	3.77				
THR 81	2.56	GLU 83	3.65				
THR 81	2.14	ALA 85	3.76				
THR 81	1.83	GLN 109	3.62				
VAL 82	2.99	GLN 109	3.73				
GLU 83	2.94	LEU 113	3.67				
GLU 83	2.44	LEU 113	3.52				
GLU 83	3.15						
GLU 83	1.91						
GLU 83	3.46						
GLY 84	3.40						
ALA 85	2.70						

With PYYYWKELAQM

Hydrogen Bonds	Distance H-A (Å)	Hydrophobic Interactions	Distance (Å)	Salt Bridge	Distance (Å)	Pi-stacking	Distance (Å)
GLU 83	3.13	ALA 85	3.85	N/A	N/A	N/A	N/A
GLU 83	2.11	ALA 85	3.81				
GLU 83	3.44	ALA 85	3.53				
GLY 84	3.32	ALA 85	3.77				
GLY 84	2.62	ALA 85	3.84				
ALA 85	2.82	PHE 94	3.97				
ALA 85	2.15	GLN 109	3.87				
ALA 85	2.50						
GLY 86	2.33						
LYS 96	3.08						

With PIWWYWKELAQM

Hydrogen Bonds	Distance H-A (Å)	Hydrophobic Interactions	Distance (Å)	Salt Bridges	Distance (Å)	Pi-stacking	Distance (Å)
THR 81	2.86	THR 81	3.71	N/A	N/A	N/A	N/A
THR 81	2.16	GLU 83	3.90				
THR 81	2.48	GLU 83	3.51				
GLU 83	2.05	GLU 83	3.56				
GLU 83	2.45	ALA 85	3.74				
GLU 83	2.30	ALA 85	3.61				
GLU 83	2.83	ALA 85	3.68				
GLY 84	2.44						
GLY 84	2.34						
GLY 84	3.10						
ALA 85	2.60						

With PWIWYWKDPNGS

Hydrogen Bonds	Distance H-A (Å)	Hydrophobic Interaction	Distance (Å)	Salt Bridges	Distance (Å)	Pi-stacking	Distance (Å)
THR 81	3.30	ALA 85	3.69	N/A	N/A	N/A	N/A
THR 81	2.25	ALA 85	3.89				
GLU 83	3.10						
GLU 83	2.56						
GLU 83	2.28						
GLU 83	3.07						
GLU 83	1.96						
GLU 83	2.75						
GLU 83	2.53						
GLY 84	2.98						
GLY 84	3.66						
GLY 84	2.18						
ALA 85	2.91						
ALA 85	1.94						

With EQALMPWIWYWKDPNGS

Hydrogen Bonds	Distance H-A (Å)	Hydrophobic Interactions	Distance (Å)	Salt Bridges	Distance (Å)	Pi-stacking	Distance (Å)
TYR 39	1.82	TYR 39	3.65	N/A	N/A	N/A	N/A
THR 44	3.58	TYR 39	3.29				
THR 44	2.49	TYR 39	3.41				
LYS 80	3.16	TYR 39	3.49				
LYS 80	2.77	THR 44	3.73				
LYS 80	2.66	THR 44	3.68				
LYS 80	2.49	LYS 80	3.68				
LYS 80	2.62	THR 81	3.90				
THR 81	3.02	THR 81	3.79				
GLU 83	2.73	THR 81	3.70				

With ELAQMPYYYWKDPNGS

Hydrogen Bonds	Distance H-A (Å)	Hydrophobic Interactions	Distance (Å)	Salt Bridges	Distance (Å)	Pi-stacking	Distance (Å)
THR 81	1.88	THR 81	3.77	N/A	N/A	N/A	N/A
THR 81	2.33	GLU 83	3.65				
THR 81	2.66	GLU 83	3.85				
THR 81	2.58	GLU 83	3.95				
THR 81	1.98	ALA 85	3.80				
THR 81	3.19	ALA 85	3.69				
VAL 82	3.51						
VAL 82	3.74						
VAL 82	2.75						
GLU 83	2.81						
GLU 83	2.70						
GLU 83	1.92						
GLU 83	1.88						
GLU 83	3.20						
GLU 83	2.89						
GLU 83	3.40						

GLU 83	2.84
GLU 83	3.62
GLU 83	2.54
GLU 83	2.03
GLU 83	2.35
GLU 83	2.11
GLY 84	2.36
GLY 84	3.12
GLY 84	3.25

With ELAQMPIWWYWKDPNGS

Hydrogen Bonds	Distance H-A (Å)	Hydrophobic Interactions	Distance (Å)	Salt Bridges	Distance (Å)	Pi-stacking	Distance (Å)
THR 81	2.40	THR 81	3.97	N/A	N/A	N/A	N/A
THR 81	2.18	THR 81	3.99				
THR 81	2.67	GLU 83	3.63				
THR 81	2.57	GLU 83	3.75				
GLU 83	2.33						
GLU 83	1.99						
GLU 83	2.30						
GLU 83	2.71						
GLU 83	3.55						
GLU 83	2.80						
GLY 84	3.56						
GLY 84	2.48						
ALA 85	2.06						
ALA 85	2.49						
ALA 85	3.34						

With DPNGSPYYYWKELAQM

Hydrogen Bonds	Distance H-A (Å)	Hydrophobic Interaction	Distance (Å)	Salt Bridges	Distance (Å)	Pi-Cation Interactions	Distance (Å)
LYS 80	2.53	LYS 80	3.63	N/A	N/A	LYS 80	5.27
LYS 80	2.35	LYS 80	3.86			LYS 80	4.22
THR 81	2.61	LYS 80	3.92				
THR 81	2.65	THR 81	3.62				
THR 81	2.60	THR 81	3.67				
THR 81	2.51	GLU 83	3.58				
THR 81	2.30						
THR 81	3.15						
GLU 83	2.78						
GLU 83	2.05						
GLU 83	2.14						
GLU 83	3.22						
GLU 83	2.98						
GLU 83	2.77						
GLY 84	3.43						
GLY 84	3.24						
GLY 84	2.54						

With DPNGSPIWWYWKELAQM

Hydrogen Bonds	Distance H-A (Å)	Hydrophobic Interaction	Distance (Å)	Salt Bridges	Distance (Å)	Pi-stacking	Distance (Å)
LYS 80	2.46	THR 44	3.70	LYS 80	3.69	N/A	N/A
LYS 80	2.74	LYS 80	3.86	LYS 80	5.02		
LYS 80	2.90	LYS 80	3.53				
THR 81	2.65	THR 81	3.65				
GLU 83	2.88	THR 81	3.86				
		THR 81	3.78				

With ELAQMGPEGPMGLEDPNGS

Hydrogen Bonds	Distance H-A (Å)	Hydrophobic Interactions	Distance (Å)	Salt Bridges	Distance (Å)	Pi-stackings	Distance (Å)
LYS 80	3.11	THR 81	3.45	N/A	N/A	N/A	N/A
THR 81	2.17	THR 81	3.63				
THR 81	2.04	GLU 83	3.81				
THR 81	2.24						
THR 81	2.52						
GLU 83	3.16						
GLU 83	3.01						
GLU 83	3.54						
GLU 83	3.65						
GLU 83	3.21						
GLU 83	3.17						
GLU 83	2.23						
GLU 83	3.09						
GLU 83	2.09						
GLY 84	3.45						

With EQALMGFYGPTEDPNGS

Hydrogen Bonds	Distance H-A (Å)	Hydrophobic Interactions	Distance (Å)	Salt Bridges	Distance (Å)	Pi-stacking	Distance (Å)
TYR 39	3.07	TYR 39	3.63	LYS 80	2.93	N/A	N/A
THR 44	2.72	TYR 39	3.56	LYS 80	4.05		
THR 44	2.40	LYS 80	3.82				
THR 44	2.71	THR 81	3.68				
THR 44	2.62						
GLU 46	3.66						
GLU 46	2.35						
GLU 46	2.20						
LYS 80	2.66						
LYS 80	2.48						
LYS 80	2.59						
THR 81	3.08						
THR 81	2.77						
THR 81	2.37						
GLU 83	2.38						
GLU 83	2.42						
GLU 83	2.51						

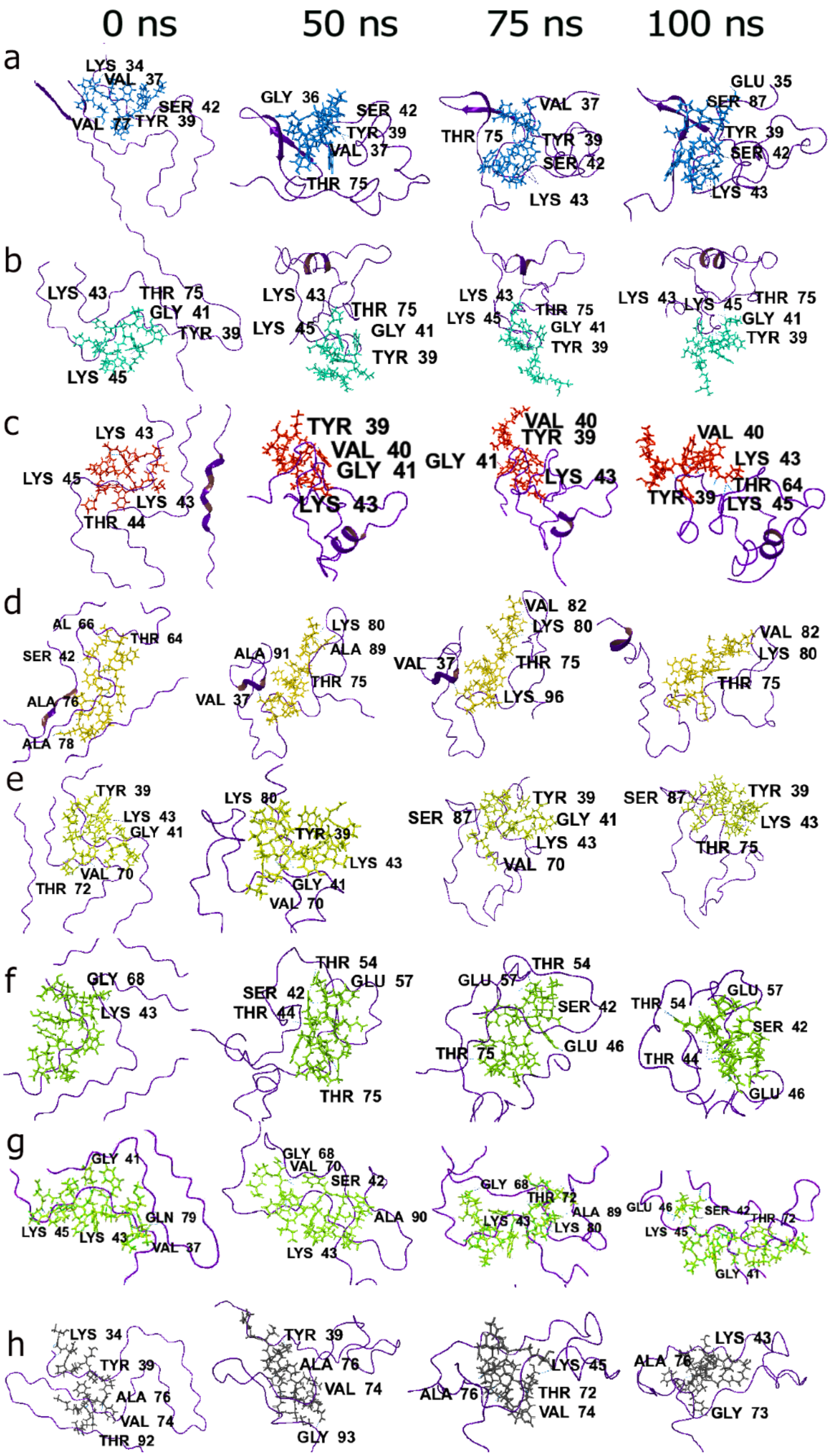

Figure S1. Trajectory snapshots of peptide complexes with ASyn derived from LBD filaments over 100 ns simulations (a) PIWWYWKDPNGS; (b) PYYYWKELAQM (c) PWIWYWKDPNGS; (d) ELAQMPYYYWKDPNGS; (e) ELAQMPIWWYWKDPNGS; (f) DPNGSPIWWWWYWKELAQM; (g) DPNGSPIWWYWKELAQM; (h) EQALMGFYGPTEDPNGS

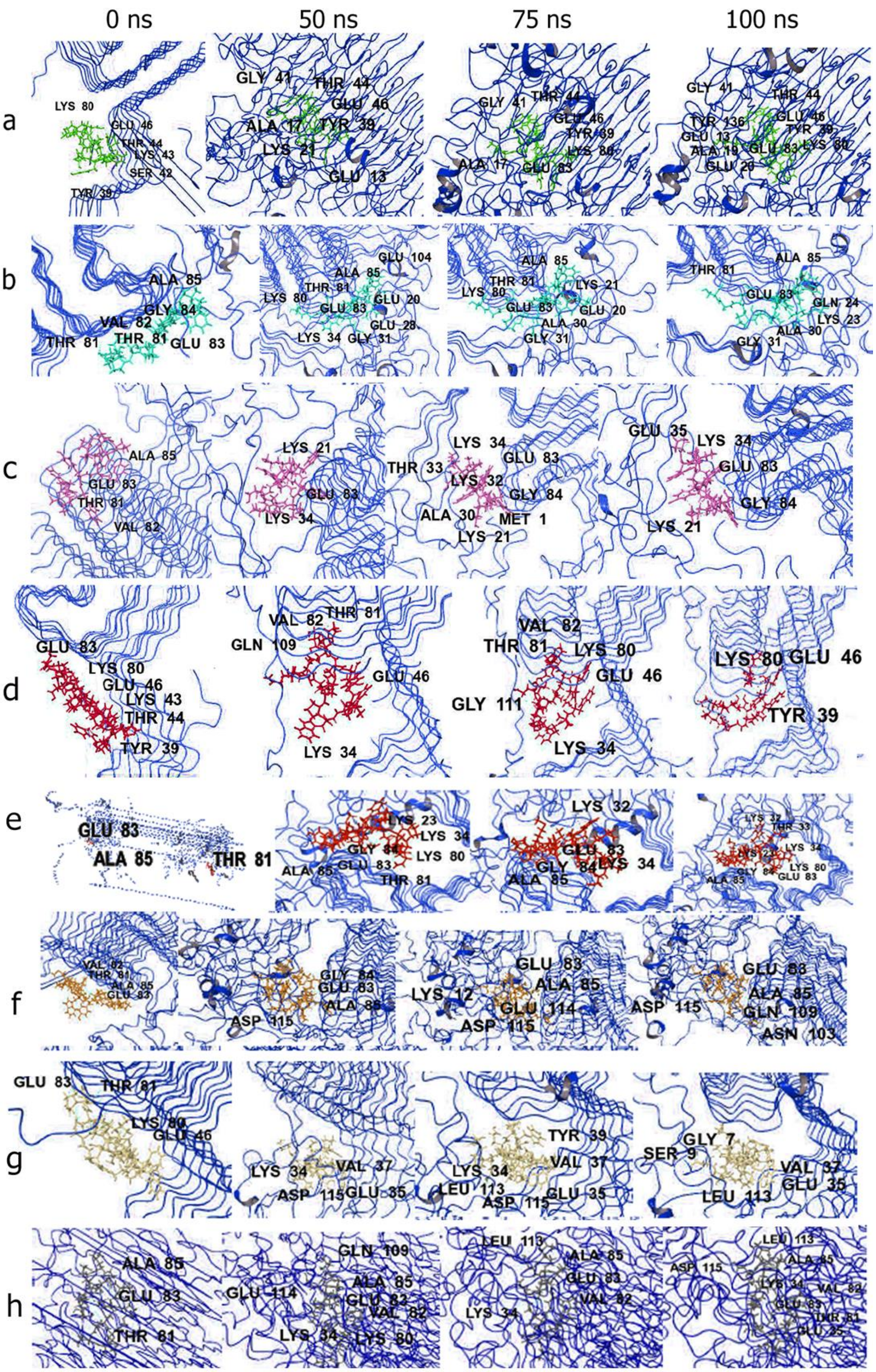

Figure S2. Trajectory snapshots of peptide complexes with ASyn fibrils over 100 ns simulations (a) PYYYWKDPNGS; (b) PIWWYWKDPNGS; (c) PWIWYWKDPNGS; (d) EQALMPWIWYWKDPNGS; (e) ELAQMPYYYWKDPNGS; (f) ELAQMPIWWYWKDPNGS; (g) DPNGSPIWWWYWKELAQM; (h) ELAQMGPEGPMGLEDPNGS.

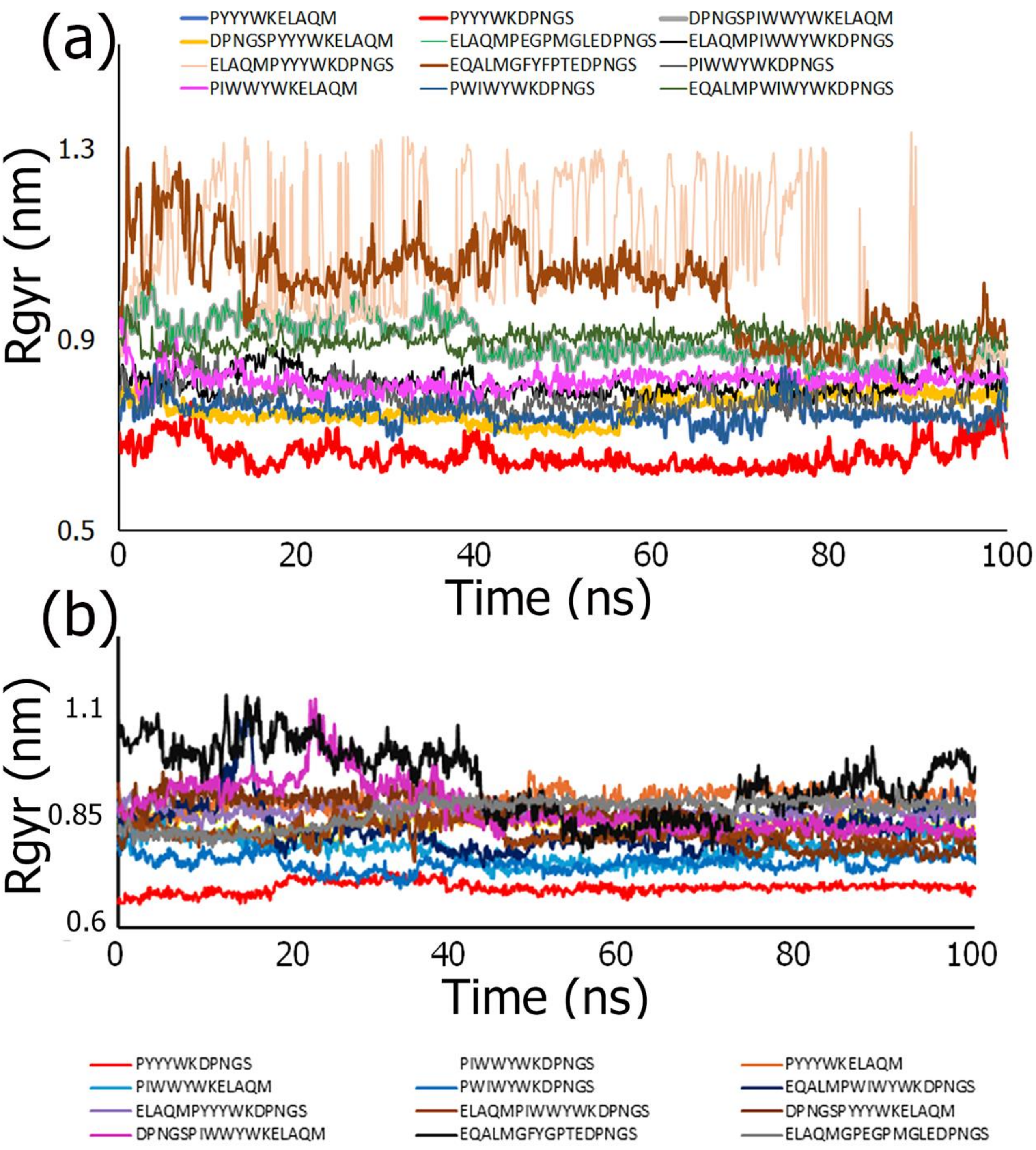

Figure S3. Radius of gyration studies of designed peptides with (a) ASyn filament derived from LBD; (b) Pathogenic ASyn fibrils.
